# Supplementary material for: Paired single-cell multi-omics data integration with Mowgli
Source: Nat Commun. 2023 Nov 24;14:7711. doi: 10.1038/s41467-023-43019-2 (PMC10673889; doi:10.1038/s41467-023-43019-2)
Supplement: Supplementary file 1 — Supplementary Information [file 41467_2023_43019_MOESM1_ESM.pdf]

Supplementary Information for "*Paired single-cell multi-omics data integration with Mowgli*"

The Supplementary Information is organized as follows:

- Page 2 of this PDF: Supplementary Table 1
- Page 3 of this PDF: Supplementary Table 2
- Pages 4 to 19 of this PDF: Supplementary Figures 1 to 16
- Pages 20 to 26 of this PDF: Supplementary Note 1
- Pages 27 to 43 of this PDF: Supplementary Note 2
- Page 44 of this PDF: Supplementary References

Dataset name	Technology	Modalities	Organism	Tissue	Number of cells	Number of annotations	Reference
Liu	scCAT-seq	RNA, ATAC	Human	Cell lines	206	3	Liu et al. [1]
10X PBMC	10X Mutliome	RNA, ATAC	Human	PBMC	9320	14	10X Genomics
OP Multiome	10X Multiome	RNA, ATAC	Human	Bone Marrow	6137	21	Open Problems [2]
OP CITE	CITE-seq	RNA, ADT	Human	Bone Marrow	4249	35	Open Problems [2]
BM CITE	CITE-seq	RNA, ADT	Human	Bone Marrow	29803	27	Stuart et al. [3]
TEA	TEA-seq	RNA, ATAC, ADT	Human	PBMC	7084	no annotations	Swanson et al. [4]

Supplementary Table 1. List of the datasets used in this paper and their characteristics.

Method	CPU or GPU	Dataset				
		Liu	10X PBMC	OP Multiome	OP CITE	BM CITE
Mowgli	GPU (Nvidia A100)	1mn08s	25mn00s	1h42mn	19mn47s	33mn09s
MOFA+	CPU (Intel i5)	24s	17mn29s	21mn48s	3mn24s	23mn4s
Seurat	CPU (Intel i5)	3s	1mn36s	51s	32s	5mn37s
integrative NMF	CPU (Intel i5)	6s	1mn15s	1mn31s	1mn01s	3mn10s
Cobolt	GPU (Nvidia A100)	59s	27mn00s	19mn07s	13mn28s	1h16mns
Multigrate	GPU (Nvidia A100)	42s	03mn55s	3mn04s	01mn43s	7mn18s

Supplementary Table 2. Compared running times of benchmarked methods across datasets

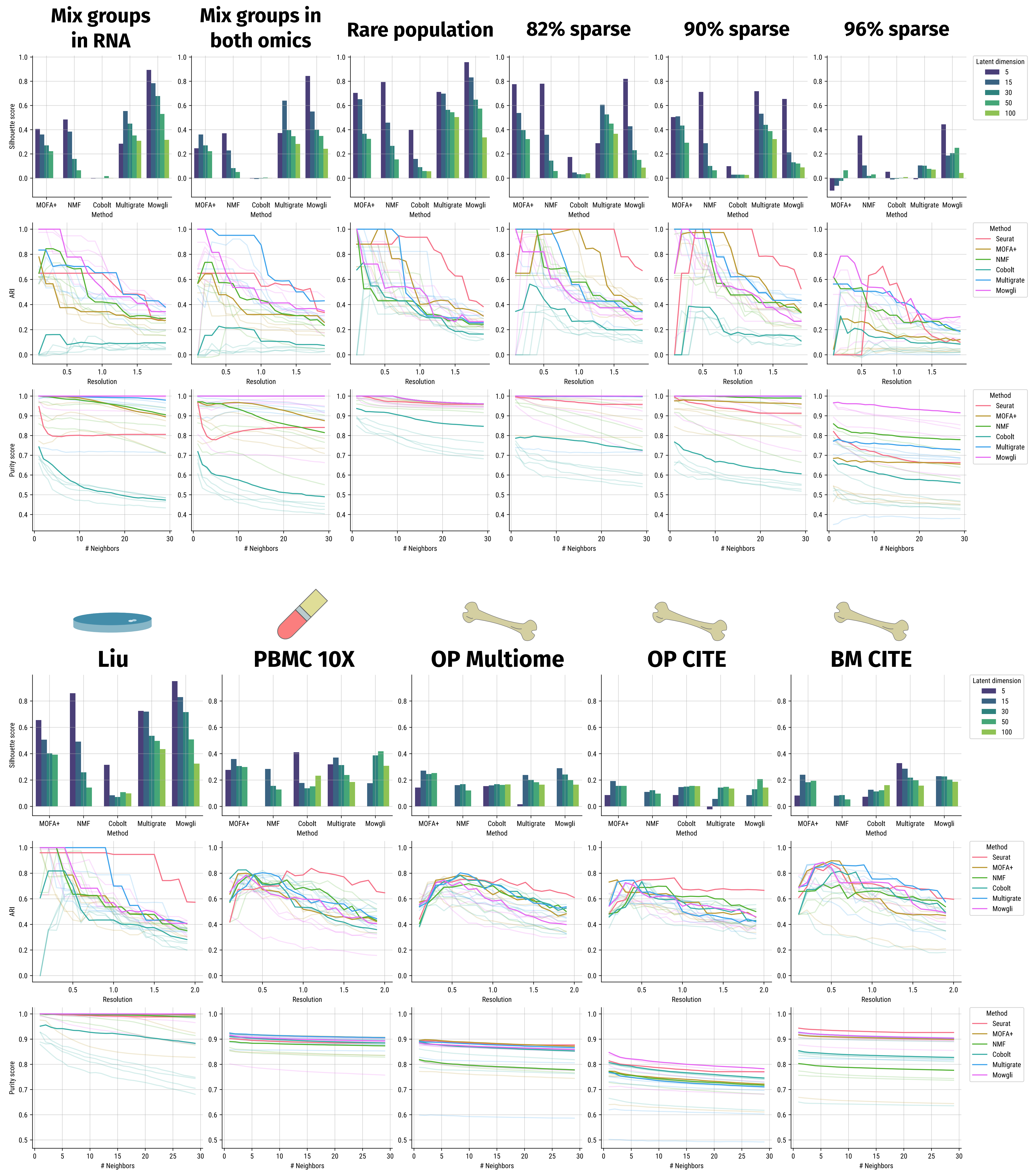

Supplementary Figure 1. Evaluation metrics across datasets and methods for the different numbers of latent dimensions. For each Adjusted Rand Index and purity score subplot, we highlight the best performing number of latent dimensions.

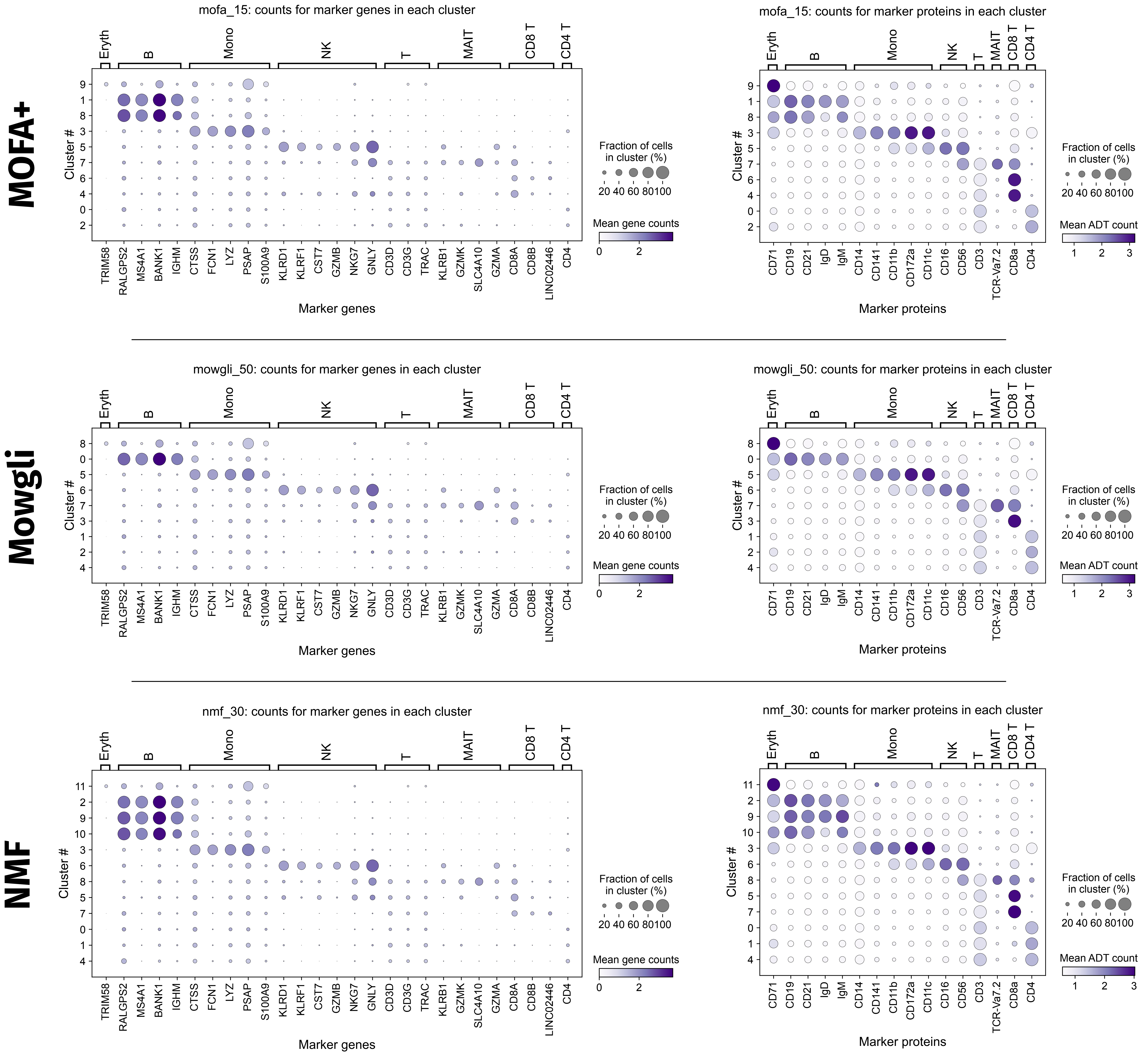

Supplementary Figure 2. Gene and protein markers for coarse annotation of TEA-seq data. Expression of gene and protein markers in the coarse Leiden clusters in the TEA-seq PBMC dataset, for Mowgli, NMF and MOFA+.

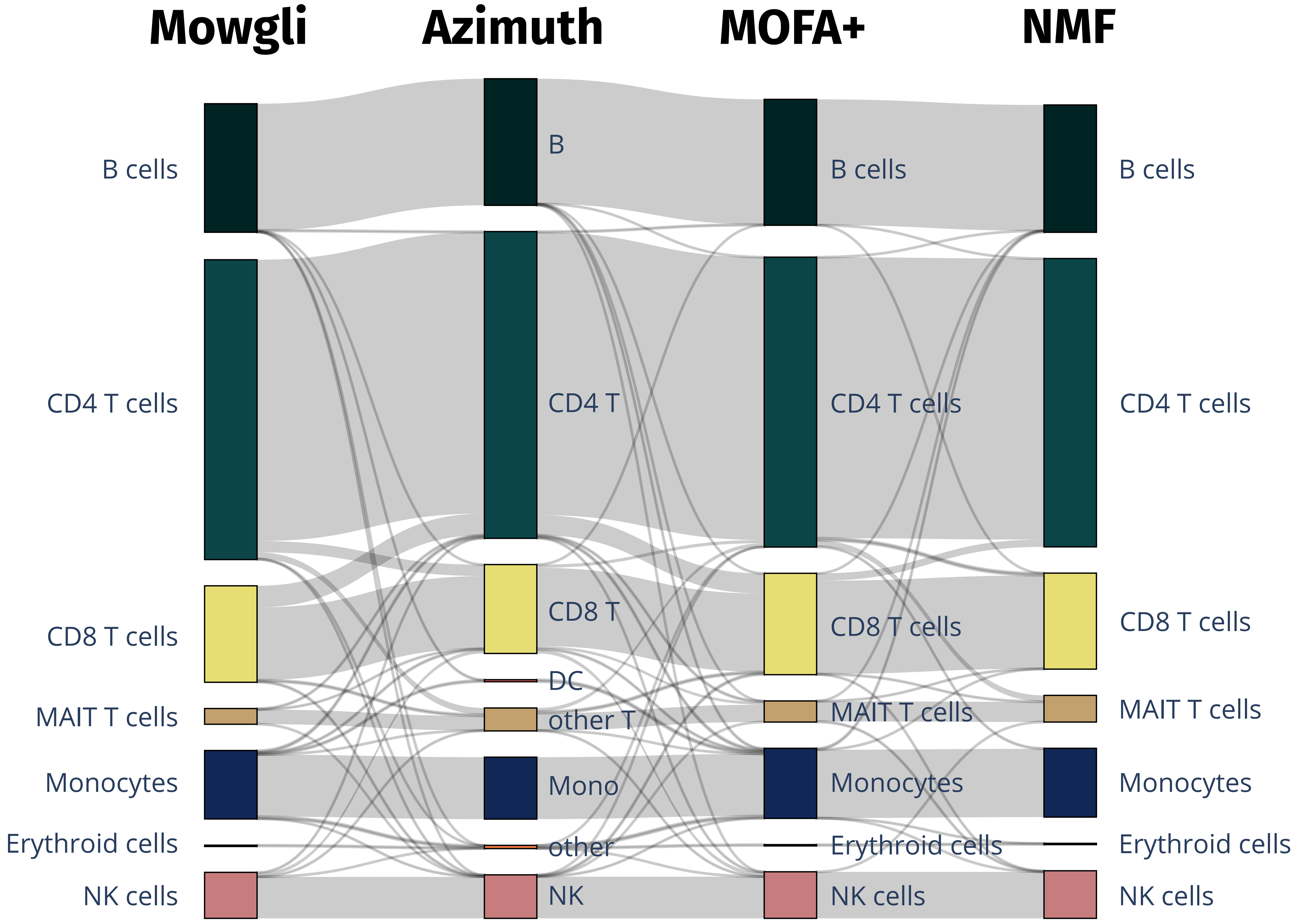

Supplementary Figure 3. Agreement between coarse annotations of TEA-seq data using different methods. We display a Sankey diagram showing the agreement between Mowgli's annotation (first), Azimuth's annotation (second), MOFA+'s annotation (third) and integrative NMF's annotation (fourth).

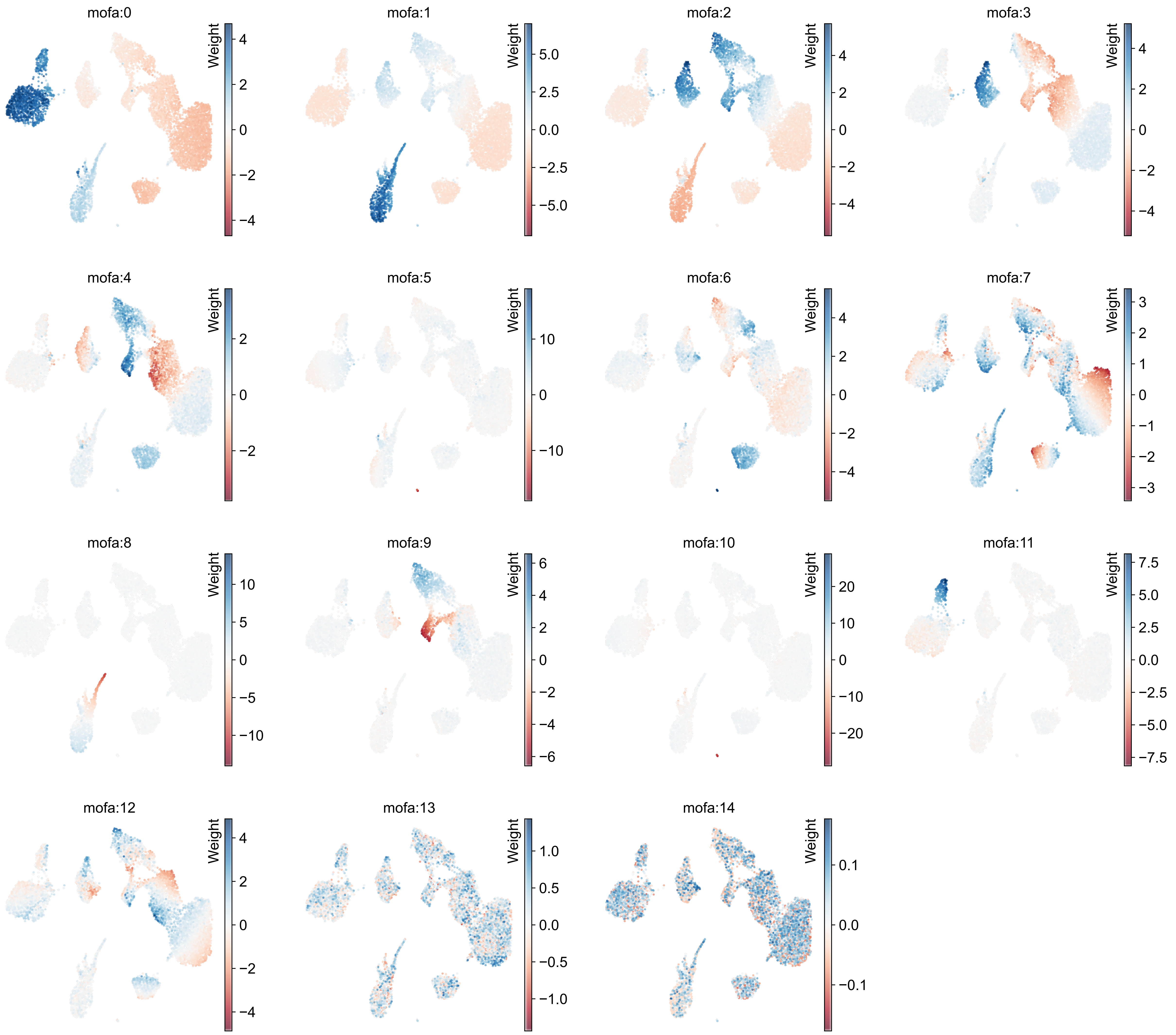

Supplementary Figure 4. MOFA+ factors across cells. For each factor of MOFA+, we display a UMAP plot of MOFA+'s embedding, each cell colored by factor weight.

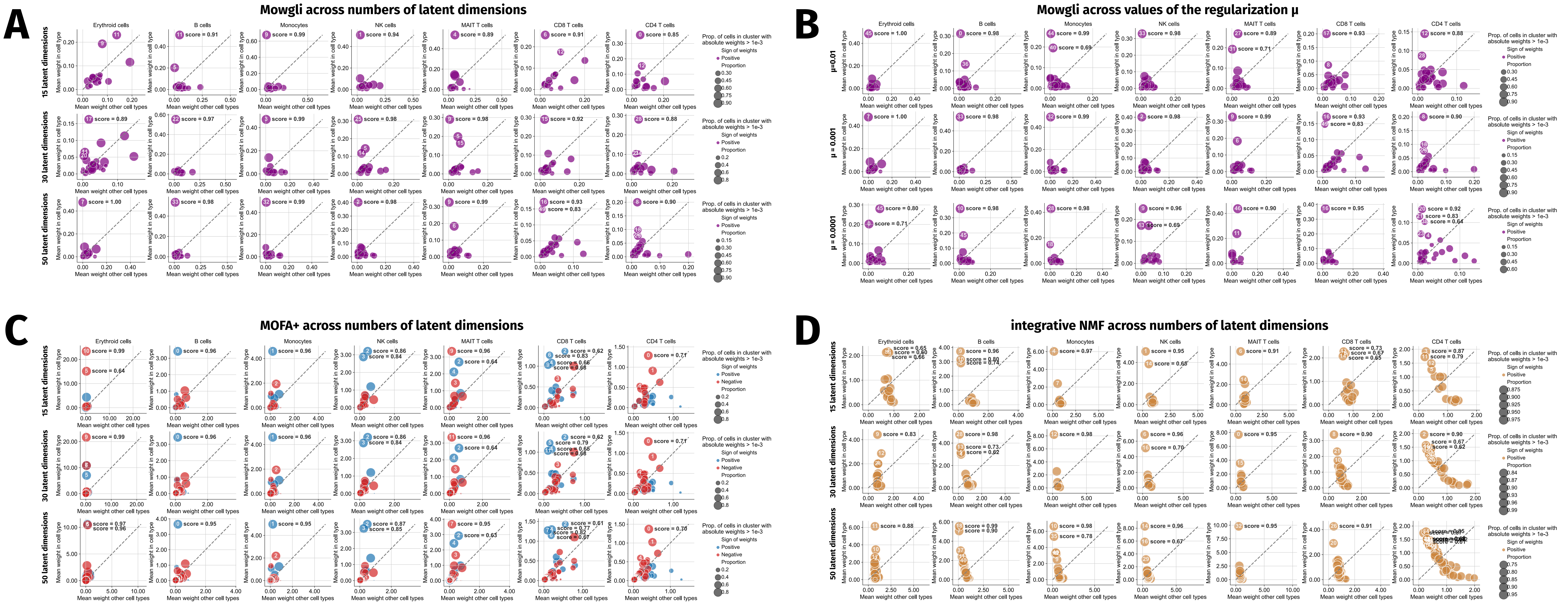

Supplementary Figure 5. Specificity scores on TEA-seq data. (A) Specificity of Mowgli's factors for 15, 30 and 50 latent dimensions. (B) Specificity of Mowgli's factors for μ equal to 0.01, 0.001, 0.0001. (C) Specificity of MOFA+'s factors for 15, 30 and 50 latent dimensions. (D) Specificity of integrative NMF for 15, 30, and 50 latent dimensions.

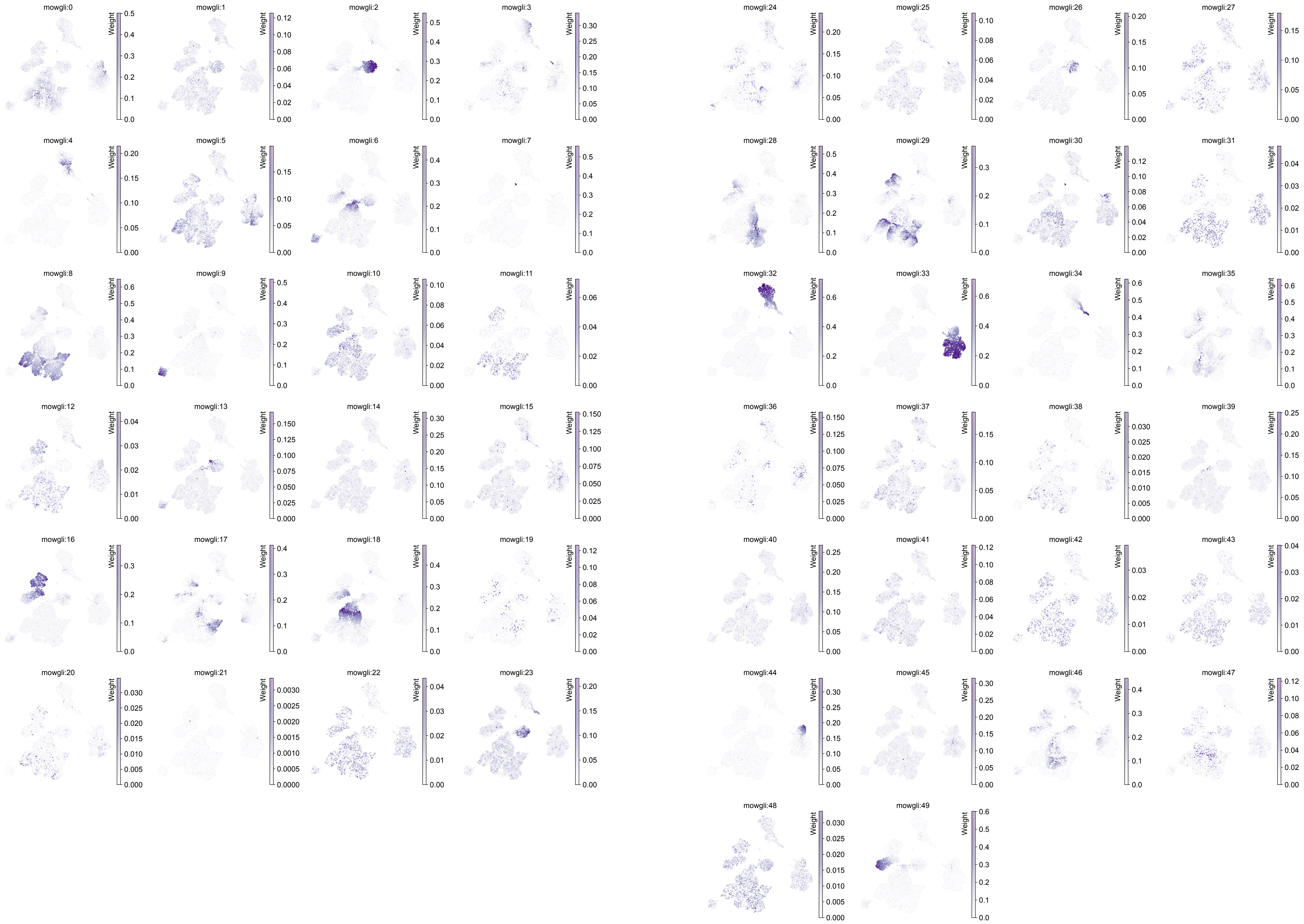

Supplementary Figure 6. Mowgli factors across cells. For each of Mowgli's factors we display a UMAP plot of Mowgli’s embedding, each cell colored by factor weight.

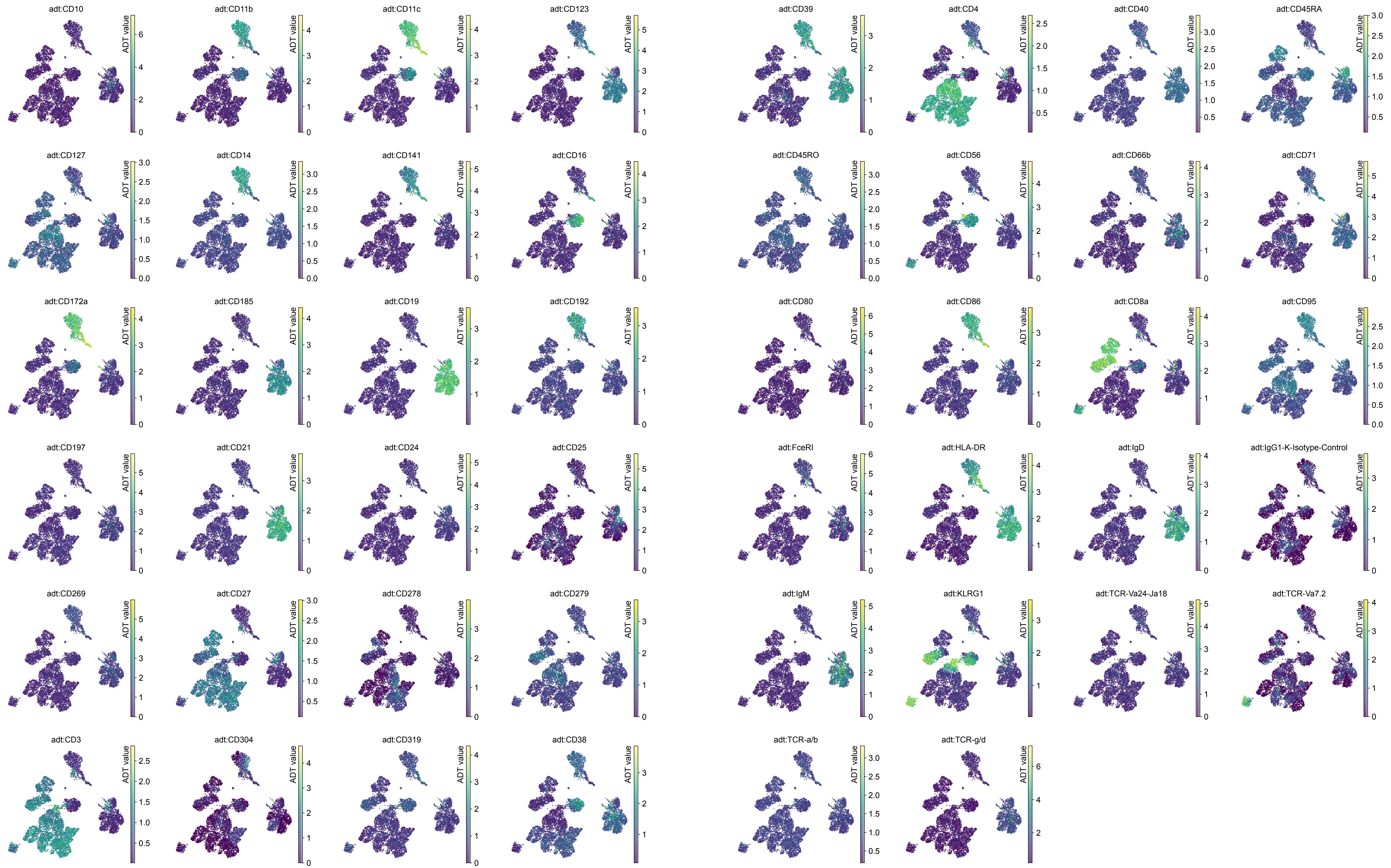

Supplementary figure 7. ADT profile and Mowgli embedding. For each protein we display a UMAP plot of Mowgli cell embeddings, colored by protein counts.

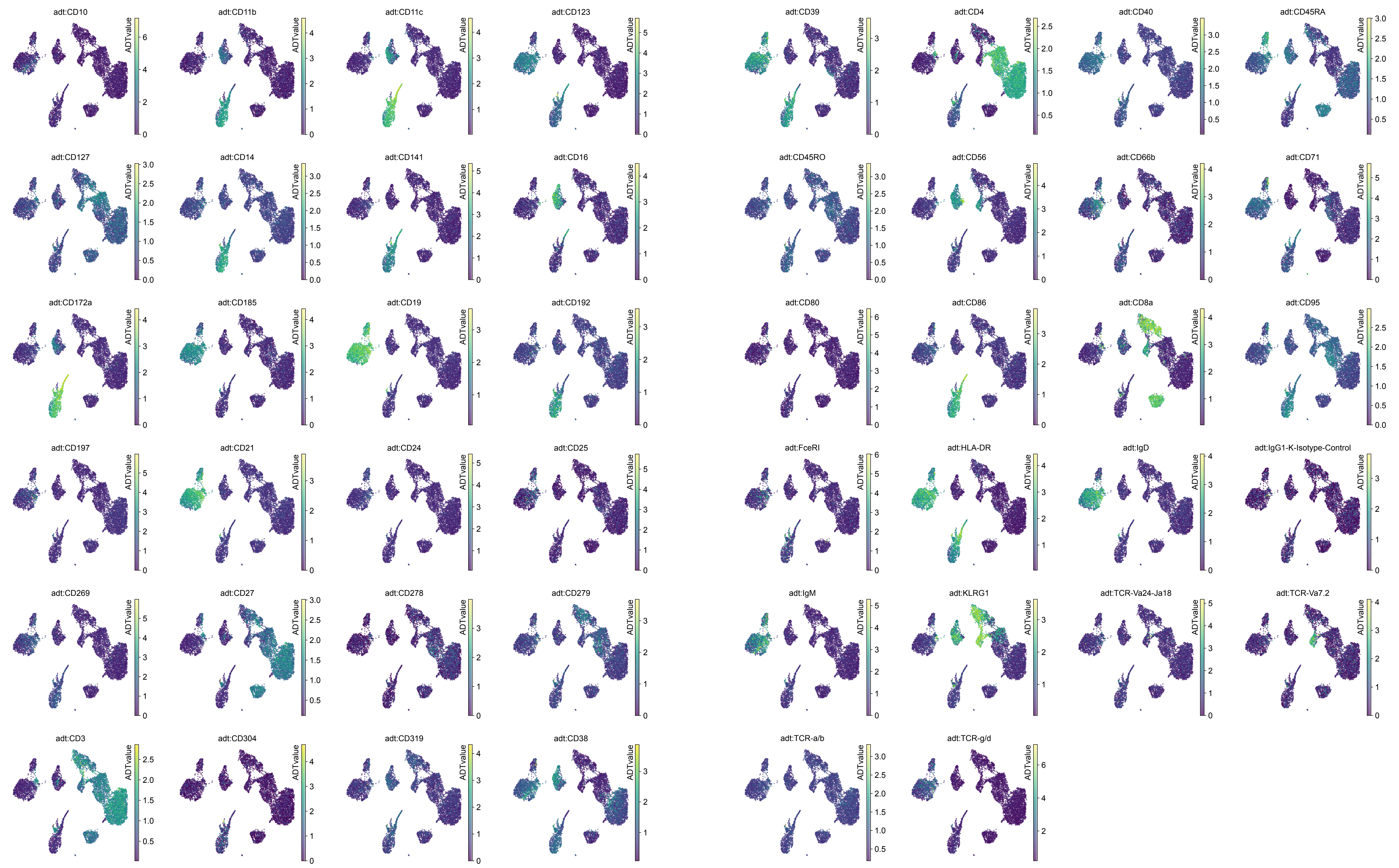

Supplementary Figure 8. ADT profile and MOFA+ embedding. For each protein we display a UMAP plot of MOFA+ cell embeddings, colored by protein counts.

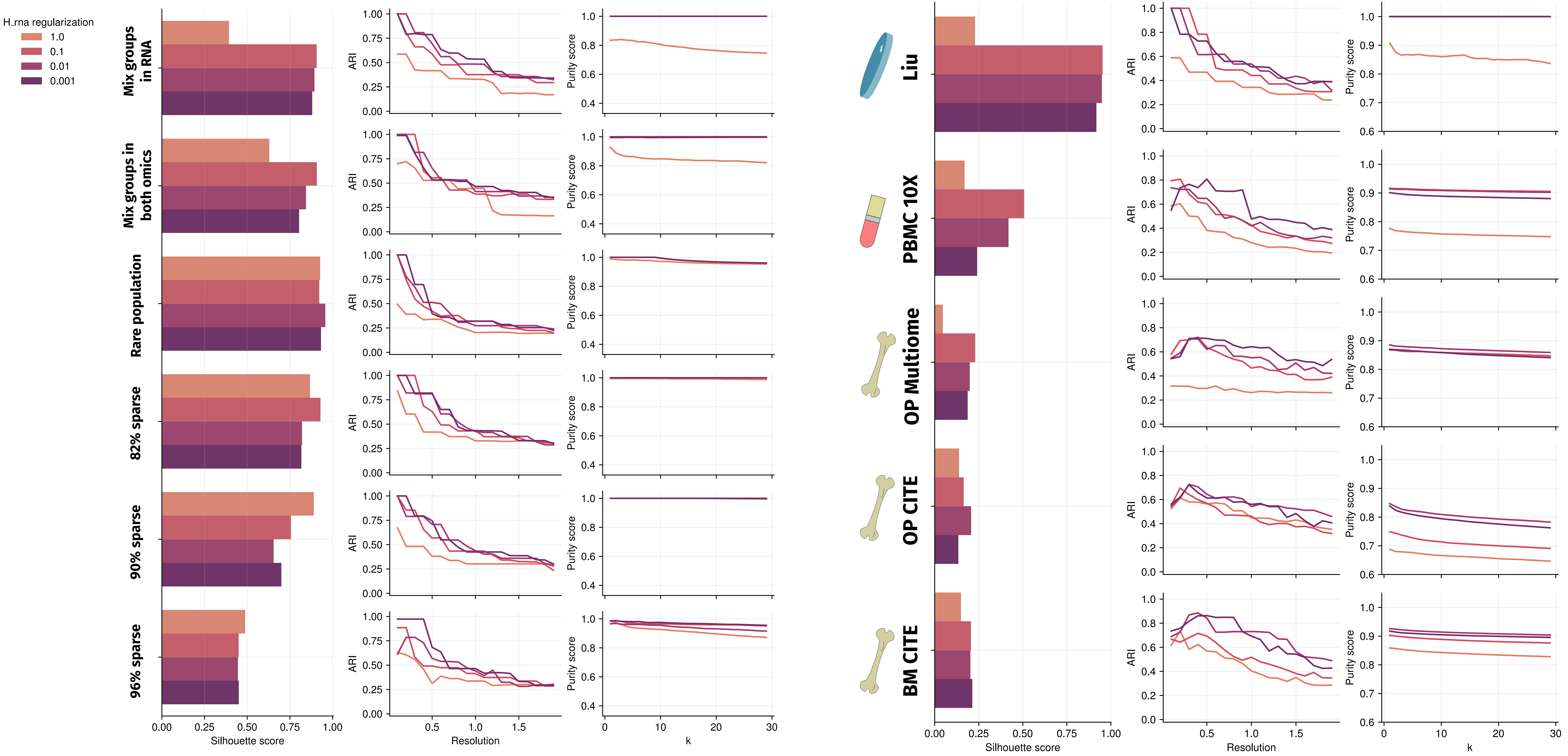

Supplementary Figure 9. Mowgli's clustering and embedding quality. Evaluated across scores, datasets and ρ_{RNA}

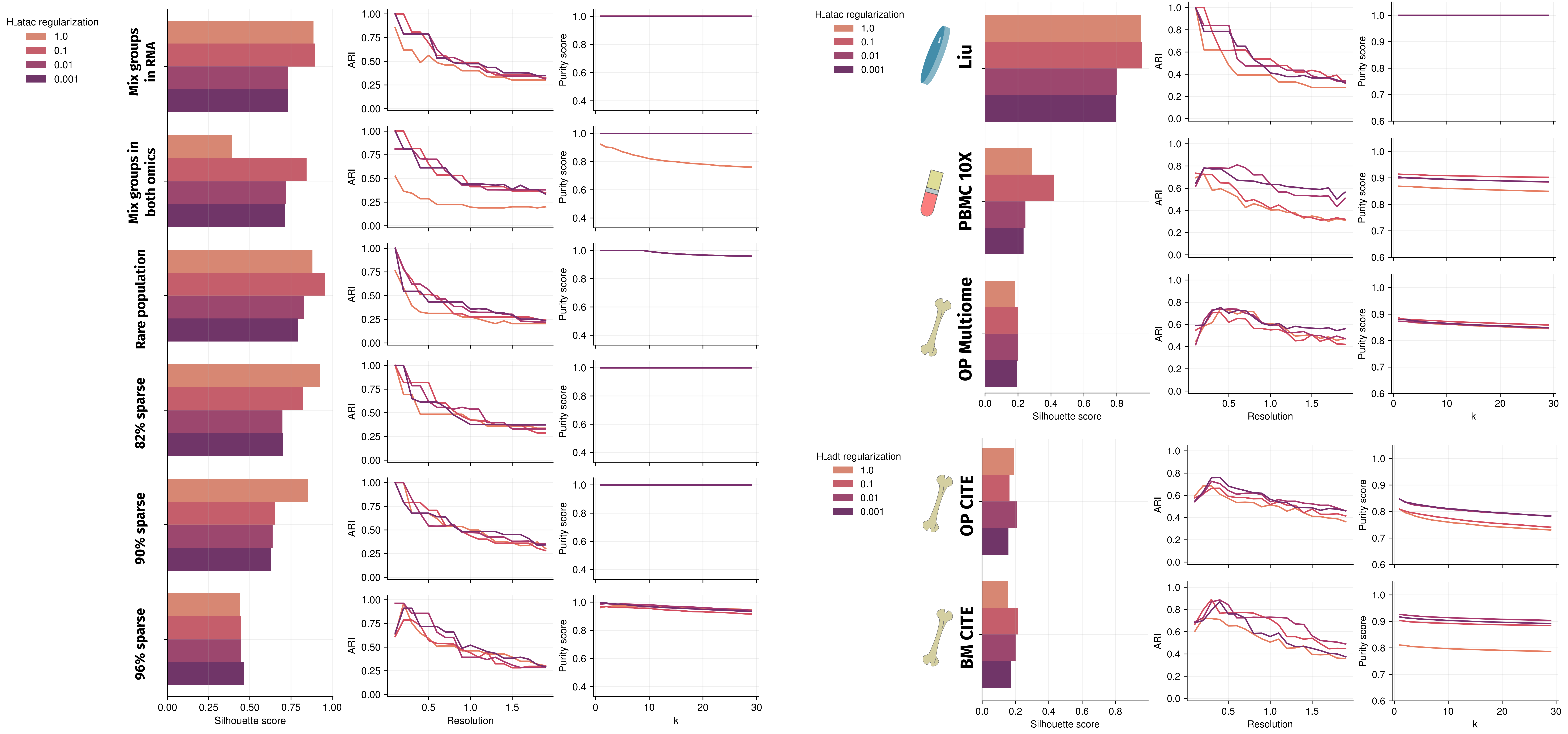

Supplementary Figure 10. Mowgli's clustering and embedding quality. Evaluated across scores, datasets and ρ_{ATAC} or ρ_{ADT}.

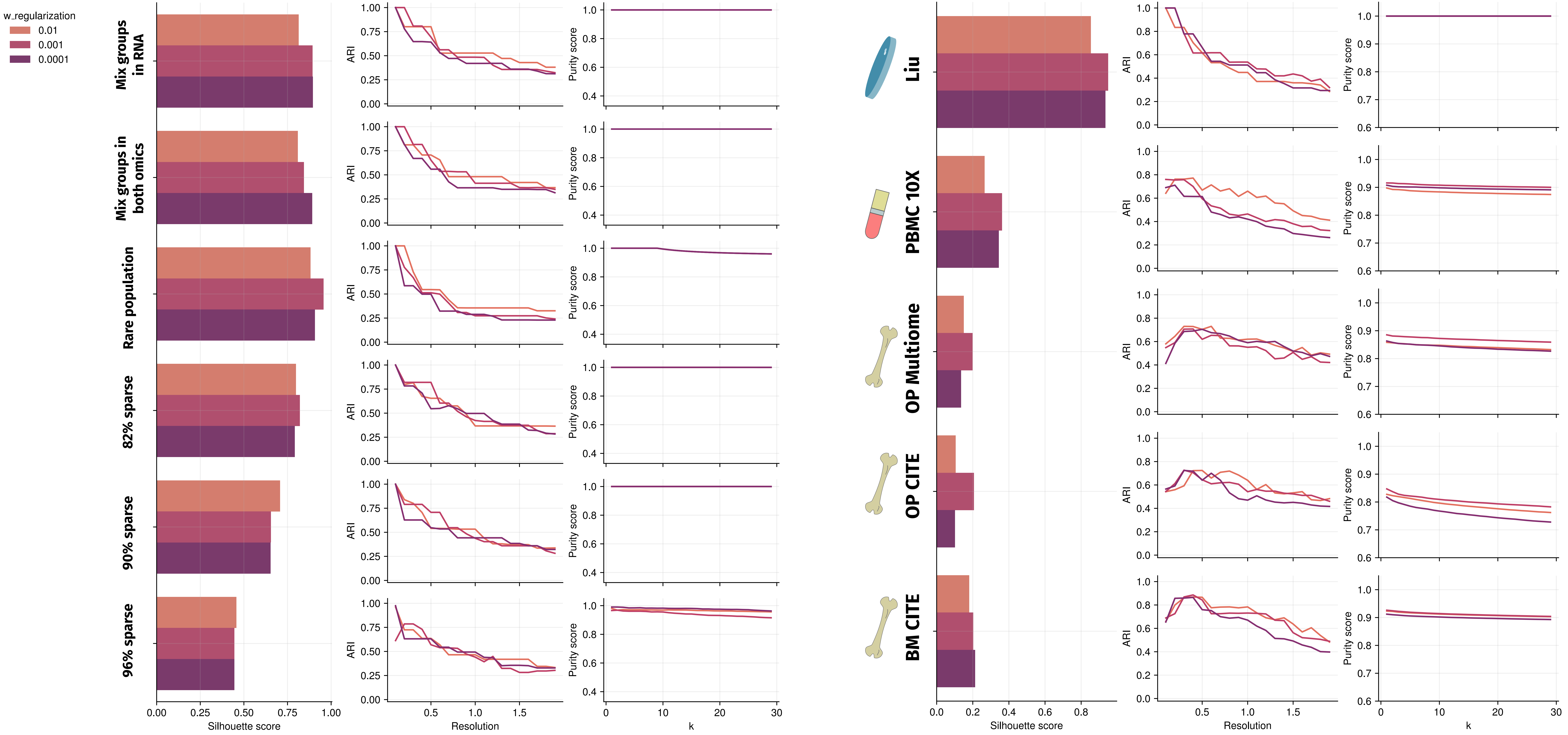

Supplementary figure 11. Mowgli's clustering and embedding quality. Evaluated across datasets, scores and μ

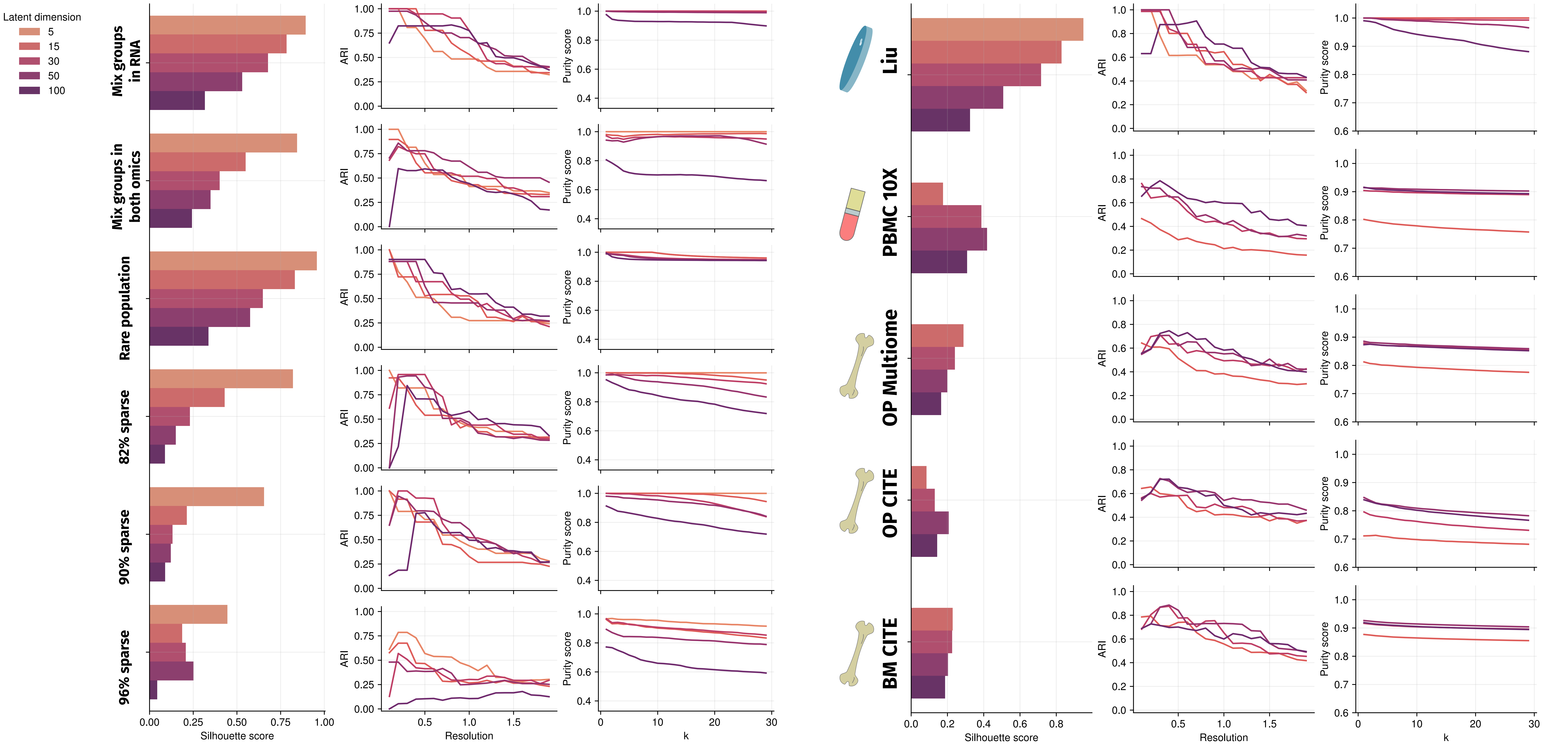

Supplementary Figure 12. Mowgli's clustering and embedding quality. Evaluated across datasets, scores and latent dimensions.

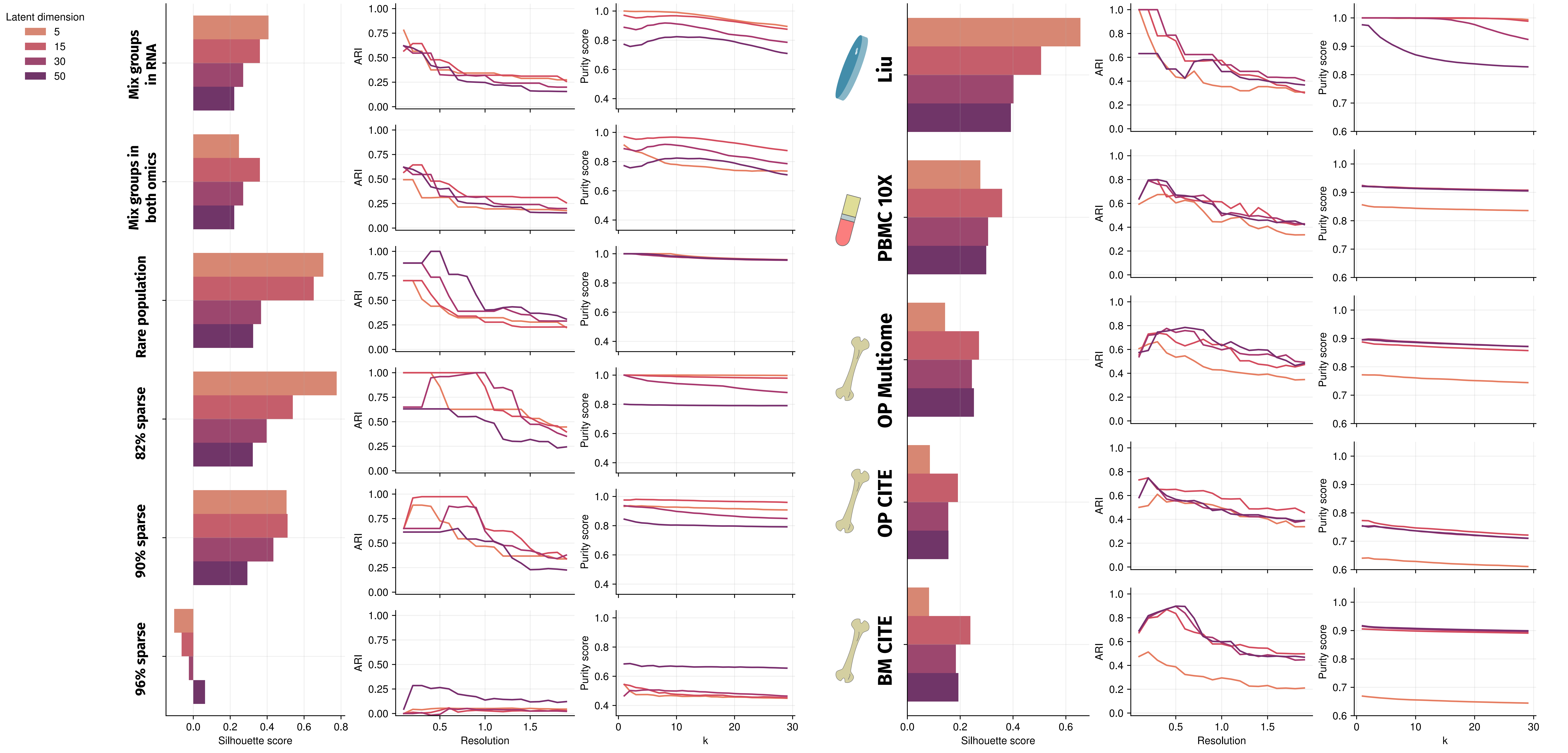

Supplementary Figure 13. MOFA+'s clustering and embedding quality. Evaluated across datasets, scores and latent dimensions.

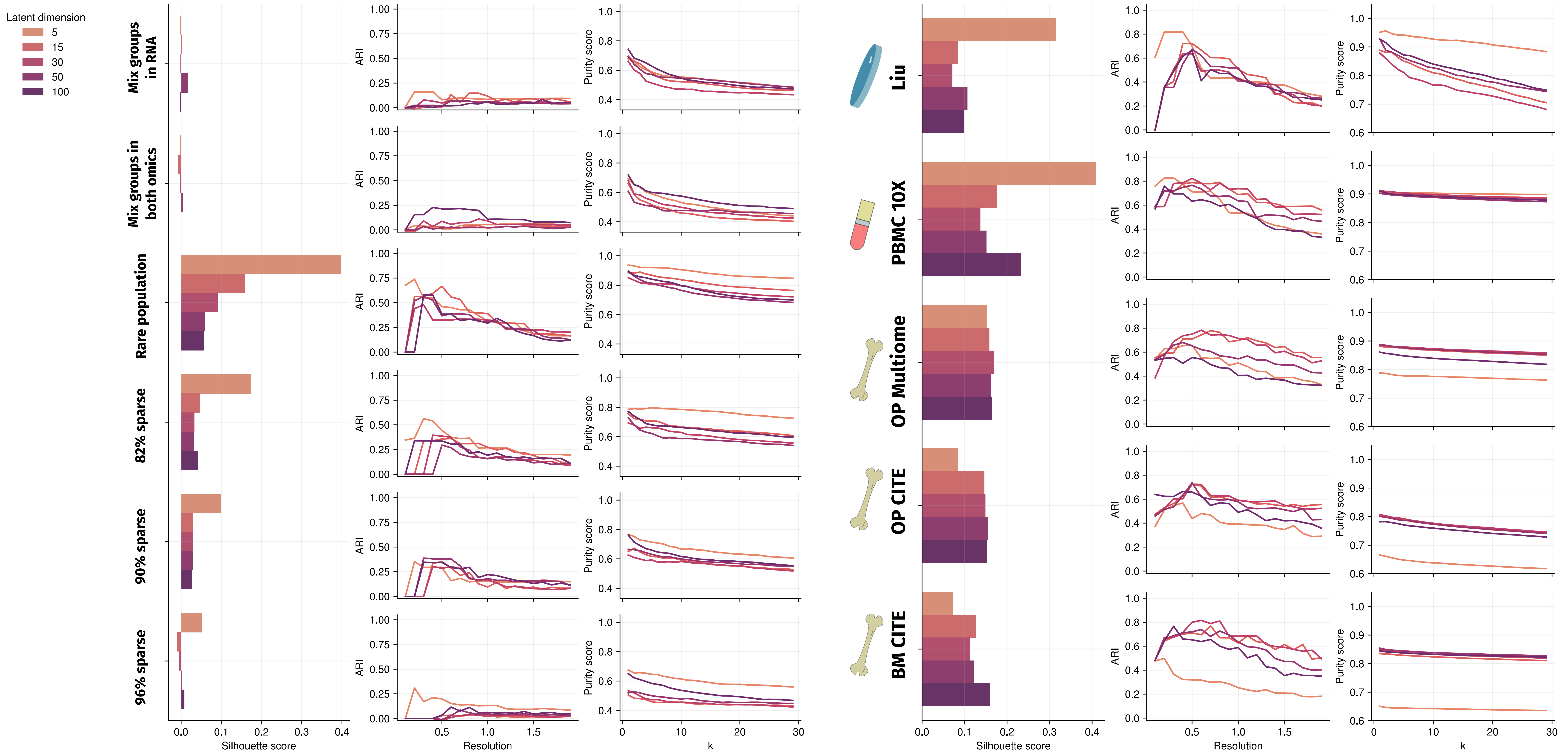

Supplementary Figure 14. Cobolt's clustering and embedding quality. Evaluated across scores, datasets and latent dimensions.

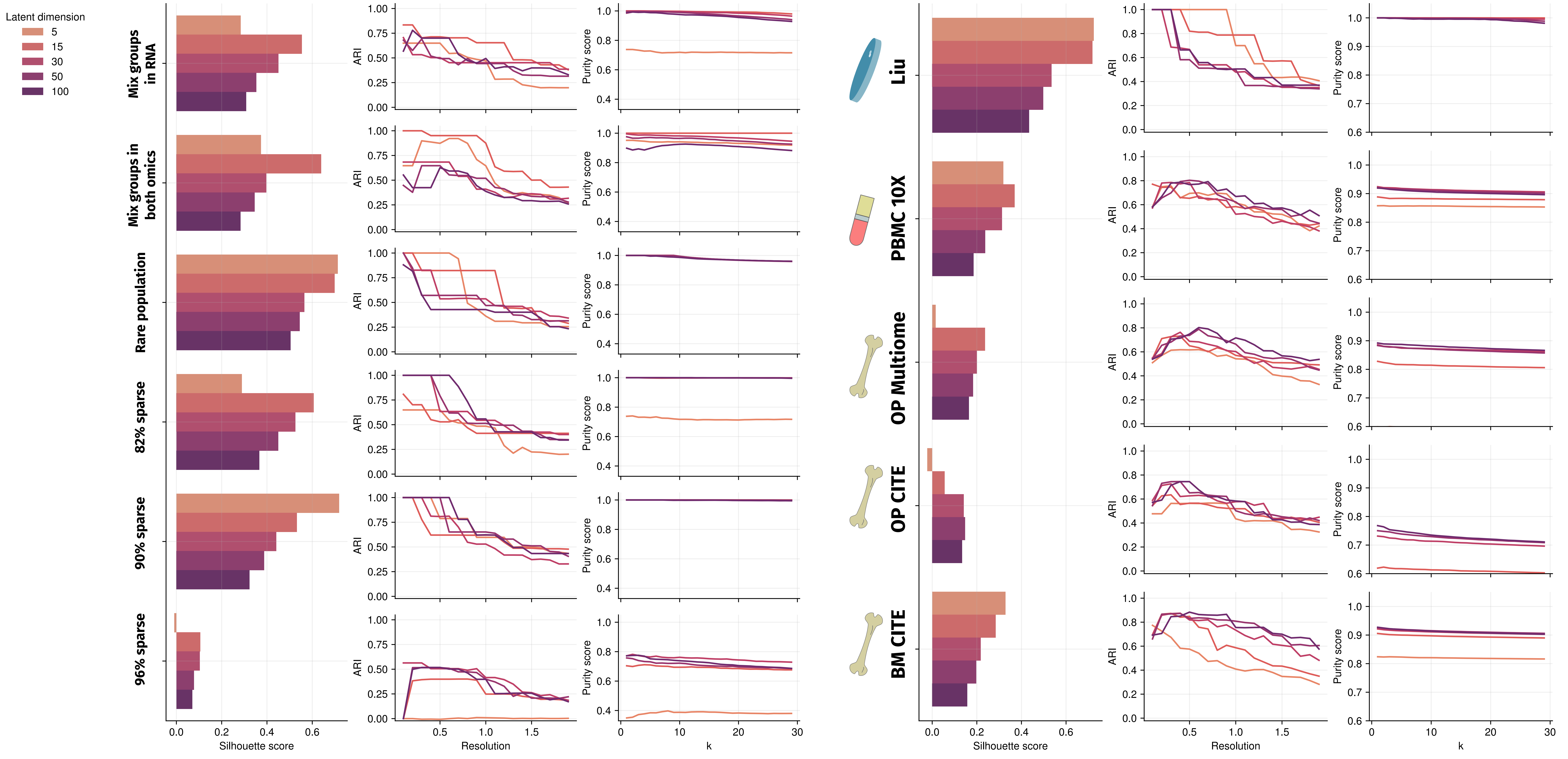

Supplementary Figure 15. Multigrate's clustering and embedding quality. Evaluated across scores, datasets and latent dimensions.

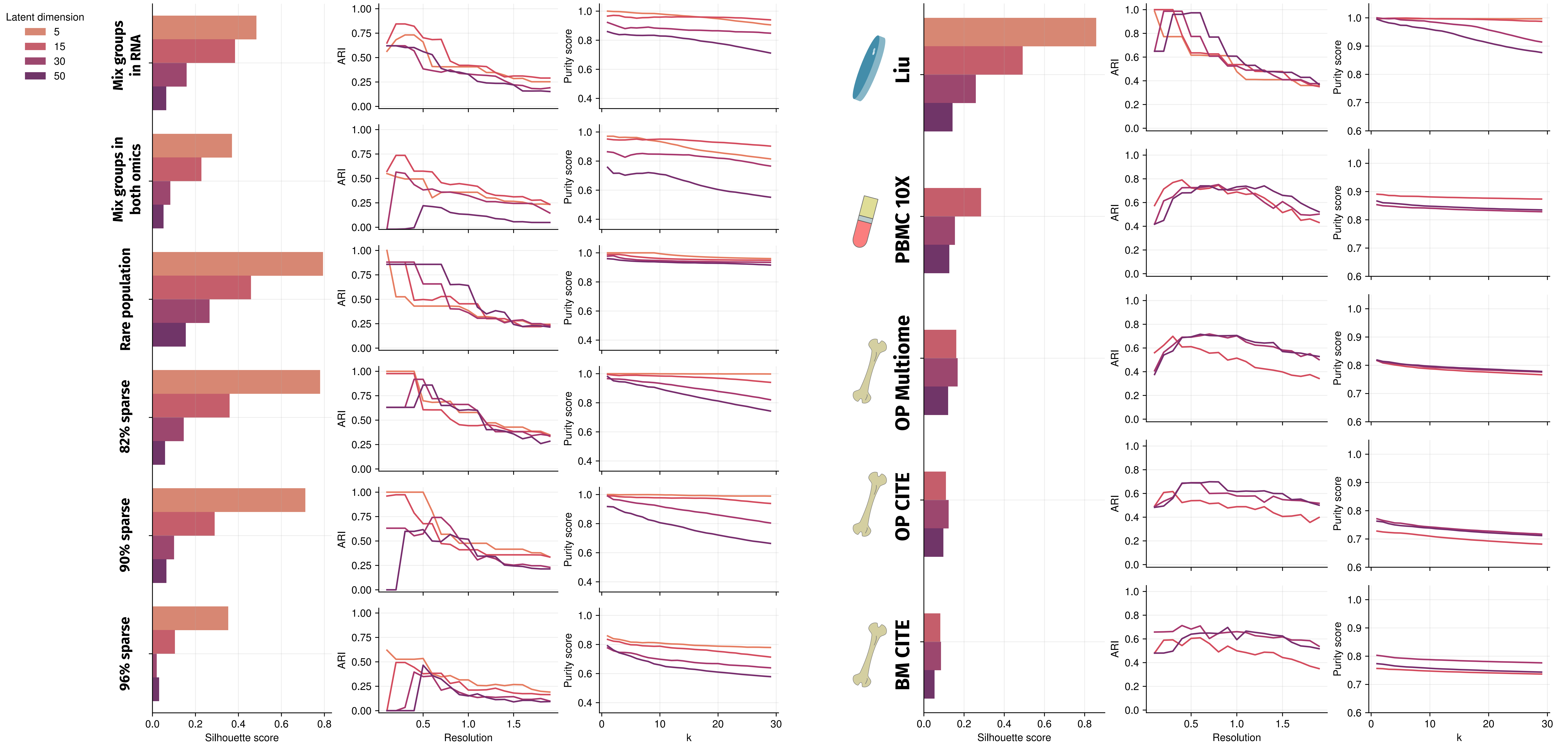

Supplementary Figure 16. Integrative NMF's clustering and embedding quality. Evaluated across scores, datasets and latent dimensions.

Supplementary Note 1 - Testing the influence of ground truth cell-type annotation on Mowgli's measured performance in real datasets

The metrics used to evaluate embedding and clustering quality in section 3 of Results rely on a ground truth cell-type annotation. Our benchmark is thus limited by the reliability and the granularity of these annotations. We illustrate these challenges using two specific edge cases which negatively affect our benchmark. First, we identify an inconsistency in the annotation of one batch in the *OP CITE* dataset. Second, we show that the granularity of the annotation in *BM CITE* limits the measured performance of Mowgli.

This supplementary note is structured as follows:

1. Influence of ground truth annotation in the *OP CITE* dataset
2. Influence of ground truth annotation in the *BM CITE* dataset

1. Influence of ground truth annotation in the *OP CITE* dataset

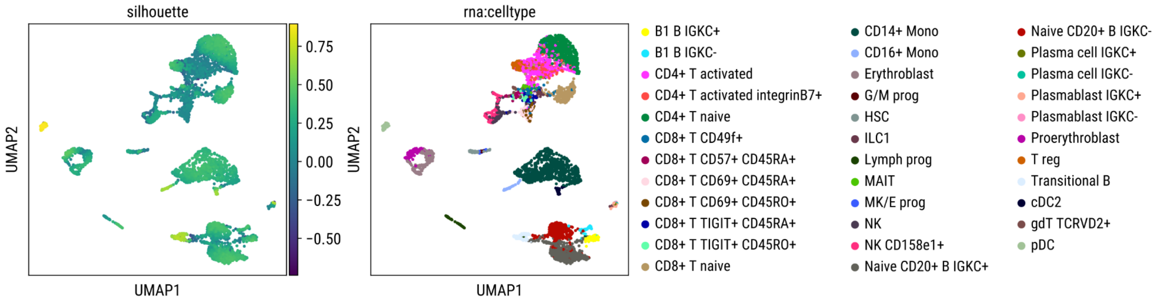

Supplementary Figure 17. Overview of Mowgli's silhouette score across cell types in *OP CITE*. UMAP plot colored by cell-wise silhouette score (left) and cell-type annotation (right) for the *OP CITE* dataset.

Supplementary Figure 17 displays the cell-wise silhouette score and the cell-type annotation for the OP CITE dataset. Subsets of CD8+ T cells have overall low silhouette scores and thus contribute negatively to Mowgli's measured performance. Among the CD8+ T cells, there are two subpopulations CD8+TIGIT+CD45RA+ and CD8+TIGIT+CD45RO+ separated by their levels of CD45RA and CD45RO.

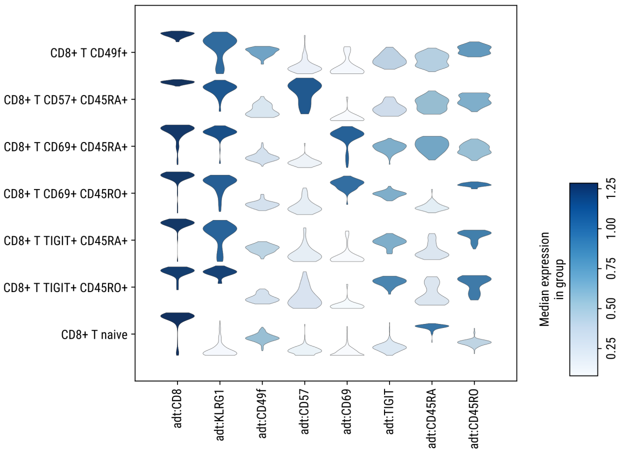

Supplementary Figure 18. Compared ADT profiles of CD8+ T cells in *OP CITE*. ADT profiles of CD8+ T cell subtypes in the *OP CITE* dataset's first batch (site 1, donor 1).

We compared the ADT profiles of CD8+ T cell subpopulations in order to investigate the reasons behind their low silhouette scores (see Supplementary Figure 18). Surprisingly, Supplementary Figure 18 shows comparable levels of CD45RO and CD45RA in the CD8+TIGIT+CD45RA+ and the CD8+TIGIT+CD45RO+ cell types.

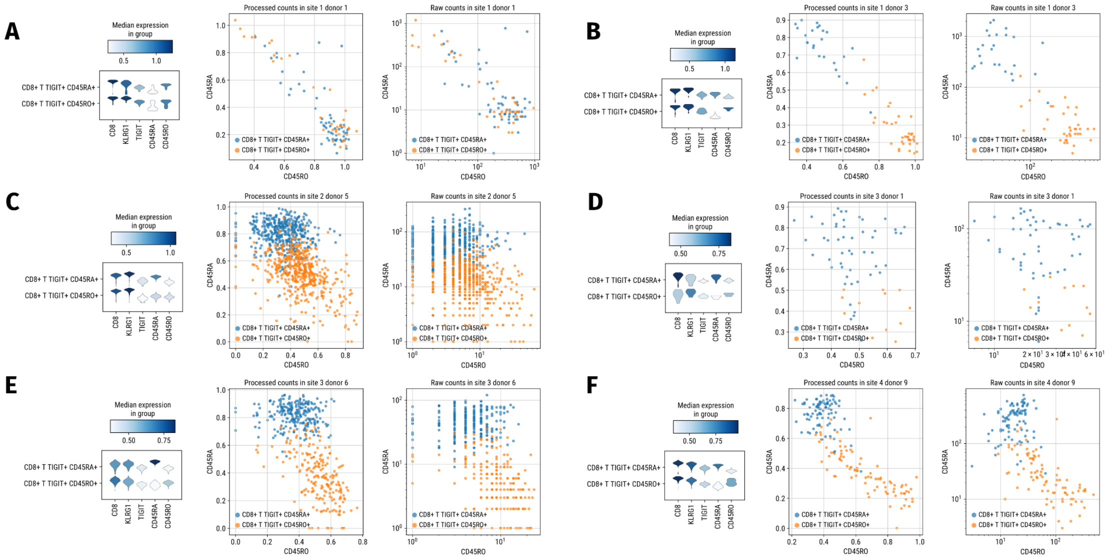

Supplementary Figure 19. Compared values of CD45RA and CD45RO in the OP CITE dataset. (A to F) Compared values of CD45RA and CD45RO in the CD8+TIGIT+CD45RA+ and the CD8+TIGIT+CD45RO+ cell types, across batches of the OP CITE dataset.

Supplementary Figure 19 displays the relative values of CD45RA and CD45RO in these two cell types as scatterplots both for normalized and raw data, and confirms that CD45RA and CD45RO do not allow distinguishing these cell types. Interestingly, this is only the case in "site 1 donor 1", the batch we used for our benchmark. Panels B to F of Supplementary Figure 19 show that in all other batches containing

these cell types, CD45RO and CD45RA patterns are undoubtedly distinct. This inconsistency in the ground truth cell-type annotation negatively affects the evaluation of the various methods in our benchmark. Indeed, cells with similar single-cell profiles but with different annotations will be embedded close together.

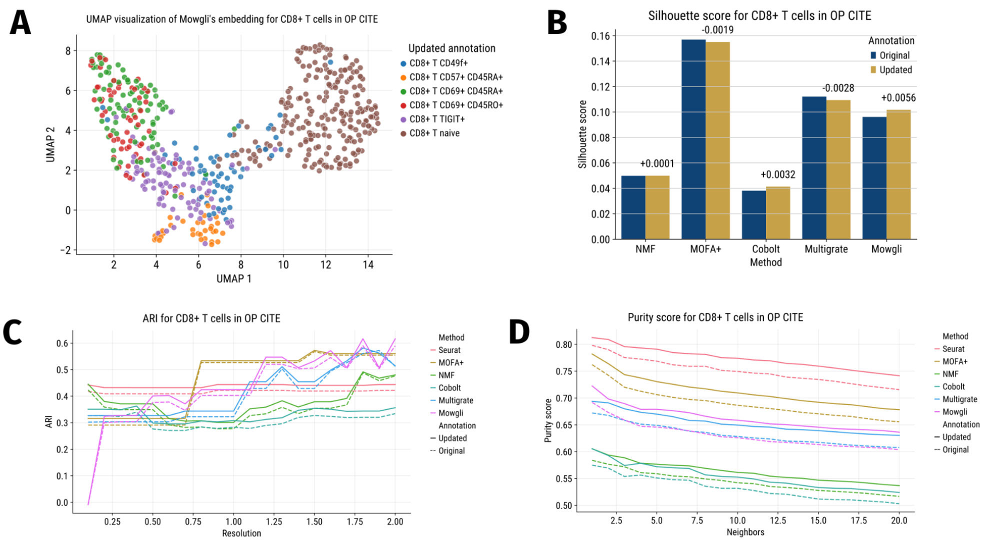

Supplementary Figure 20. Impact of imperfect ground truth annotation on evaluation metrics. (A) UMAP visualization of Mowgli's embedding for CD8+ T cells in the OP CITE data, colored by updated annotation. (B) Silhouette score for CD8+ T cells in OP CITE, before and after updating the annotation. (C) Adjusted Rand Index for CD8+ T cells in OP CITE, before and after updating the annotation. (D) Purity score for CD8+ T cells in OP CITE, before and after updating the annotation.

We investigated whether imperfect ground truth annotation impacts the evaluation metrics evenly for all methods. For this, we updated the annotation by fusing the CD8+TIGIT+CD45RA+ and CD8+TIGIT+CD45RO+ subtypes into a single CD8+TIGIT+ subtype. Panel A of Supplementary Figure 20 displays a UMAP visualization of Mowgli's embedding for the different CD8+ T cell subtypes in the *OP CITE* dataset, colored according to the updated annotation. Panel B of Supplementary Figure 20 shows silhouette scores computed across CD8+ T cells for different methods. The updated annotation leads to an increase in silhouette score for NMF, Cobolt, and Mowgli, and to a decrease in silhouette score for MOFA+ and Multigrate. Panels C and D of Supplementary Figure 20 show that the updated annotation leads to an increase in ARI and purity score for all methods. Supplementary Figure 20 shows that changes in ground truth annotation have an uneven impact on evaluation across methods and metrics. This limits the reliability of benchmarking in the presence of imperfect annotation, which should be complemented by an in-depth

biological analysis of results, as proposed in this Supplementary Note and in the Results.

2. Influence of ground truth annotation in the *BM CITE* dataset

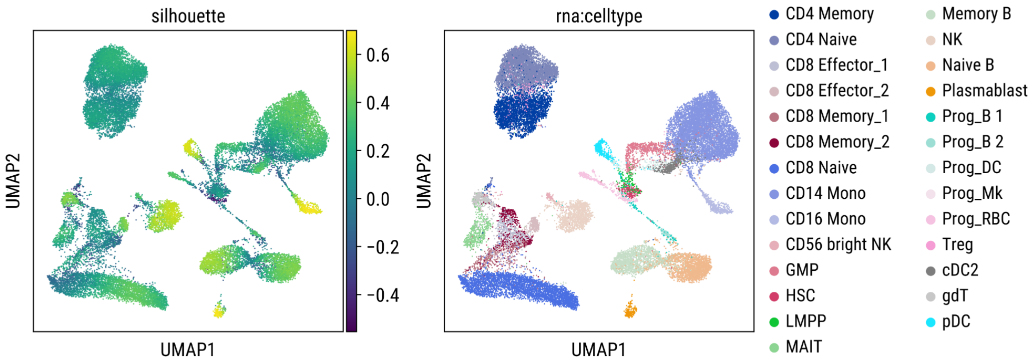

Supplementary Figure 21. Overview of Mowgli's silhouette score across cell types in *BM CITE*. Cell-wise silhouette score (left) and cell-type annotation (right) for the BM CITE dataset.

Supplementary Figure 21 displays the cell-wise silhouette score and the cell-type annotation for the BM CITE dataset. The CD8 Effector_1 and CD8 Memory_2 cell types, which were computationally derived in [5], have overall low silhouette scores and thus contribute negatively to Mowgli's measured performance.

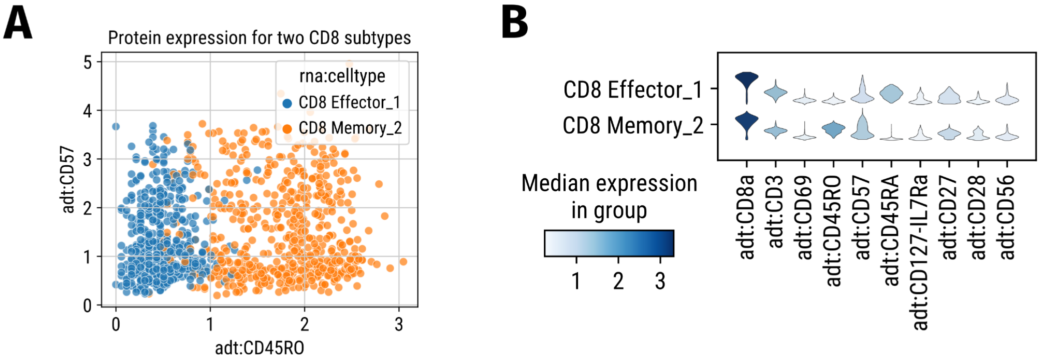

Supplementary Figure 22. Compared ADT profiles of the CD8 Effector_1 and CD8 Memory_2 populations of the BM CITE dataset. (A) Compared values of CD45RO and CD57 in the CD8 Effector_1 and CD8 Memory_2 cell types of the BM CITE dataset. (B) Compared ADT profiles of CD8 Effector_1 and CD8 Memory_2 in the BM CITE dataset.

Supplementary Figure 22 shows that CD45RO levels clearly distinguish CD8 Effector_1 and CD8 Memory_2 cells.

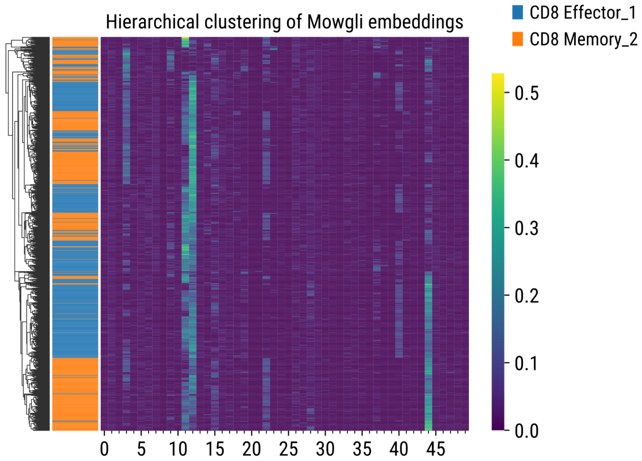

Supplementary Figure 23. Hierarchical clustering of CD8 subtypes using Mowgli embeddings. Hierarchical clustering on Mowgli's embeddings of CD8 Effector_1 and CD8 Memory_2 cells in the BM CITE dataset.

Despite this clear separation, hierarchical clustering on Mowgli's embeddings mixes CD8 Effector_1 and CD8 Memory_2 cells, which explains low silhouette scores (Supplementary Figure 23). One strong signal driving this clustering is factor 44, even though factors 22 and 40 would have allowed the clustering of CD8 Memory_2 and CD8 Effector_1 cells separately.

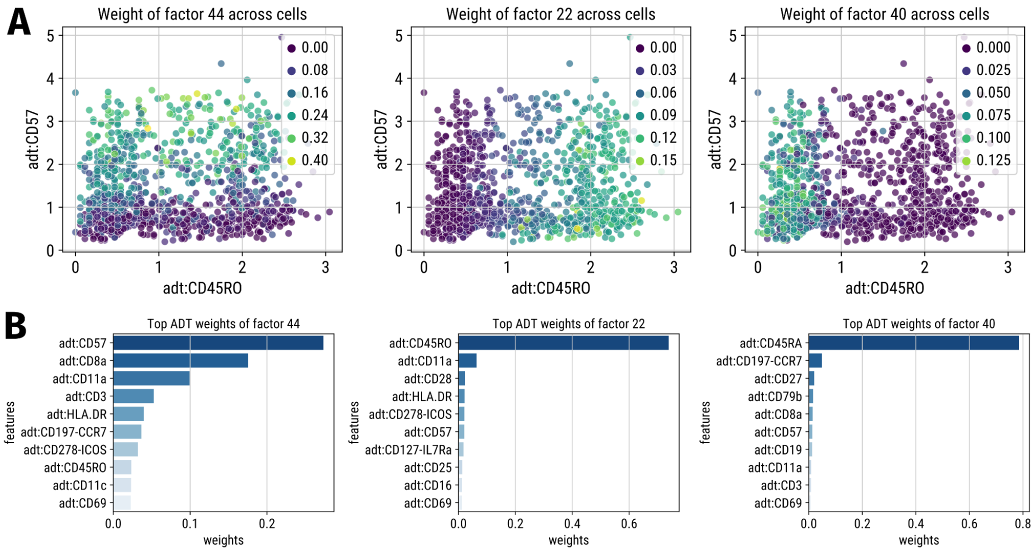

Supplementary Figure 24. Compared ADT profiles and Mowgli factor weights for the CD8 Effector_1 and CD8 Memory_2 populations of the BM CITE dataset. (A) Compared values of CD45RO and CD57 in the CD8 Effector_1 and CD8 Memory_2 cell types of the BM CITE dataset. Cells are colored by their weight of Mowgli factors 44, 22, and 40 (B) Top ADT weights in Mowgli factors 44, 22, and 40.

Supplementary Figure 24 shows that together, factors 44, 22, and 40 effectively split the CD8 Effector_1 and CD8 Memory_2 populations into four subpopulations: CD57+CD8+ Effector_1, CD57-CD8+ Effector_1, CD57+CD8+ Memory_2, and CD57-CD8+ Memory_2. While this granularity is not present in the annotation, it is mentioned in the original publication [5]. Indeed, [5] explicitly highlights the relevance of CD57 as a marker of effector cells and displays its expression in the associated Figure S2, where CD57 clearly splits CD8 Effector_1 and CD8 Memory_2 into CD57+ and CD57- subpopulations. In this example, our benchmark penalizes Mowgli even though it detects more granularity than the original annotation.

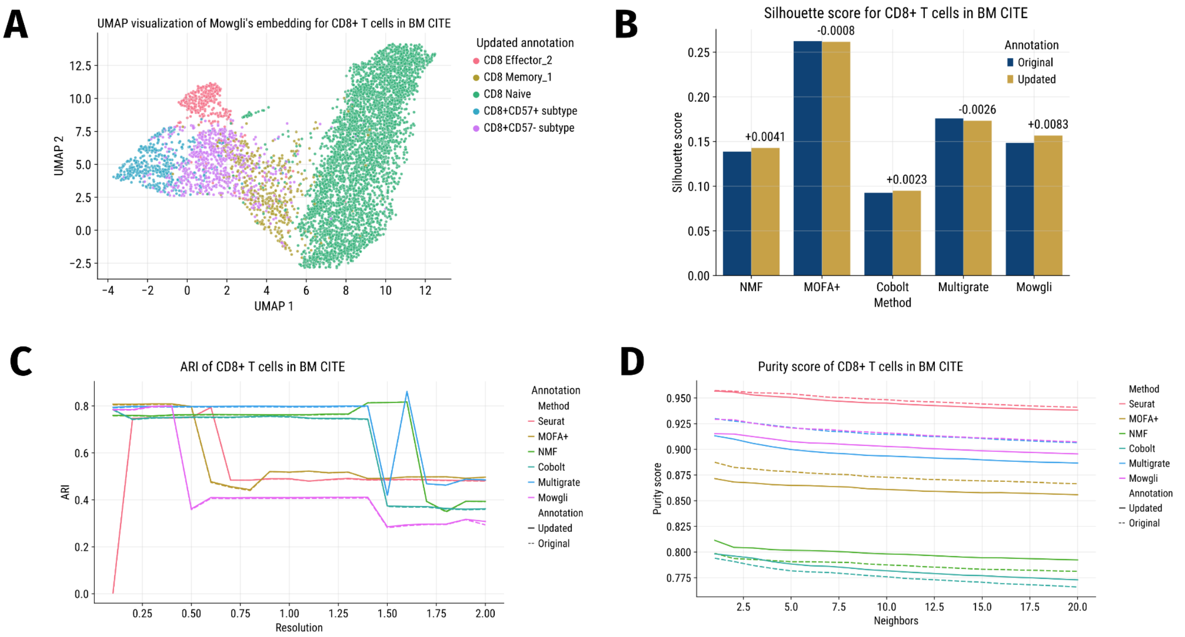

Supplementary Figure 25. Impact of imperfect ground truth annotation on evaluation metrics. (A) UMAP visualization of Mowgli's embedding for CD8+ T cells in the BM CITE data, colored by updated annotation. (B) Silhouette score for CD8+ T cells in BM CITE, before and after updating the annotation. (C) Adjusted Rand Index for CD8+ T cells in BM CITE, before and after updating the annotation. (D) Purity score for CD8+ T cells in BM CITE, before and after updating the annotation.

As in section 1 of this Supplementary Note, we investigated whether imperfect ground truth annotation impacts the evaluation metrics evenly for all methods. For this, we updated the annotation by reorganizing cells from the CD8 Effector_1 and CD8 Memory_2 subtypes into a CD57+ and a CD57- subtype. Panel A of Supplementary Figure 25 displays a UMAP visualization of Mowgli's embedding for the different CD8+ T cell subtypes in the *BM CITE* dataset, colored according to the updated annotation. Panel B of Supplementary Figure 25 shows silhouette scores

computed across CD8+ T cells for different methods. As in *OP CITE*, the updated annotation leads to an increase in silhouette score for NMF, Cobolt, and Mowgli, and to a decrease in silhouette score for MOFA+ and Multigrate. Panel C of Supplementary Figure 25 shows no influence of the updated annotation on ARI. Panel D of Supplementary Figure 25 shows that the updated annotation leads to an increase in purity score for Cobolt and NMF, and a decrease in purity score for Seurat, MOFA+, Multigrate, and Mowgli. As observed in the *OP CITE* dataset, changes in ground truth annotation have an uneven impact on evaluation across methods and metrics for the *BM CITE* dataset.

Supplementary Note 2 - Comparing the interpretability of Mowgli and MOFA+’s factors for the detection of specific subtypes in the TEA-seq PBMC dataset

To complement Figure 5b and section 5 of Results, we quantitatively analyzed factors of Mowgli associated with specific immune subpopulations in the *TEA-seq PBMC* dataset: CD56 bright NK cells, classical and nonclassical monocytes, naive CD4 T cells, plasmacytoid dendritic cells, and conventional dendritic cells.

We assessed the extent of this association through (i) the ranking of markers in the factor’s top proteins, (ii) the Pearson correlation across cells between the factor and a protein marker, and (iii) scatterplots with compared values of the factor and of protein markers. We used these same quantitative assessments to identify if MOFA+ presents factors associated with the considered subpopulations.

In nonclassical monocytes, both MOFA+ and Mowgli present a factor closely associated with the subpopulation. In the other considered subpopulations, Mowgli’s signal is more biologically relevant than MOFA+’s.

This supplementary note is structured as follows:

1. CD56 bright NK cells
2. Plasmacytoid dendritic cells
3. Naive CD4 T cells
4. Conventional dendritic cells
5. Nonclassical monocytes
6. Classical monocytes

1. CD56 bright NK cells

CD56 bright NK cells have CD56-bright and CD16-negative phenotypes. Mowgli's factor 13 is largely driven by CD56 and has a low contribution of CD16, and is thus coherent with the biological signature of CD56 bright NK cells (see Supplementary Figure 26).

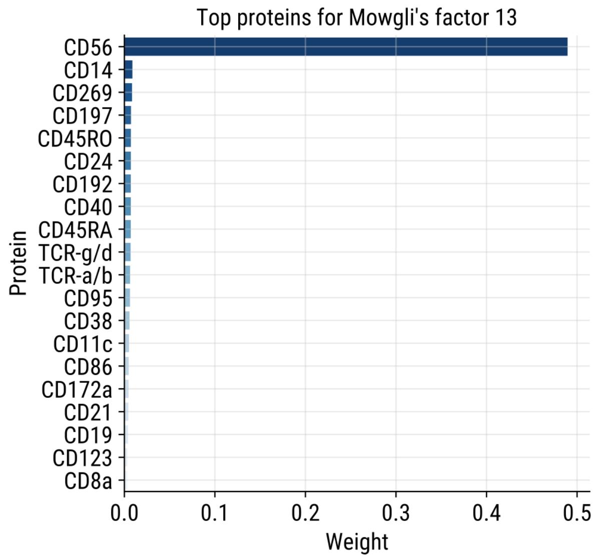

Supplementary Figure 26. Protein weights for Mowgli's factor 13. Top ADT weights for Mowgli's factor 13 in the TEA dataset.

In addition, high weights for Mowgli's factor 13 coincide with low CD16 and high CD56 (see Supplementary Figure 27).

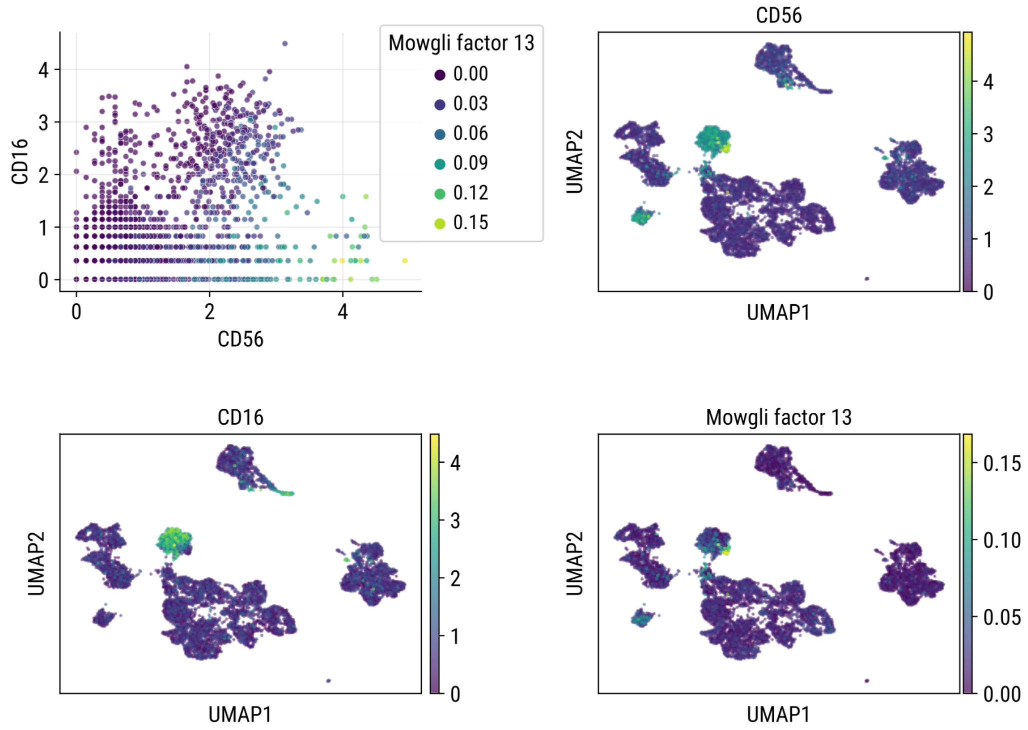

Supplementary Figure 27. Compared ADT profiles and Mowgli factor 13. Compared values of CD16, CD56, and weight of Mowgli's factor 13.

Mowgli's factor 13 is the only one with a high Pearson correlation with CD56 and a low absolute Pearson correlation with CD16 (see Supplementary Figure 28). In addition, CD56 ranks first in factor 13’s top genes, which is the best rank across factors.

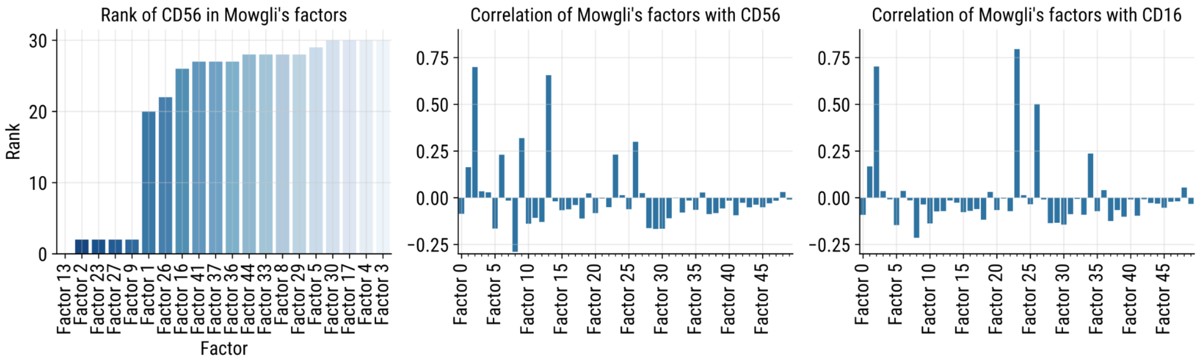

Supplementary Figure 28. Correlation between CD56, CD16, and Mowgli’s factors. Compared rank of CD56 in Mowgli’s factors, and correlation of CD56 and CD16 with Mowgli’s factors.

By contrast, MOFA+ has no factors that are both highly correlated with CD56 and uncorrelated with CD16 (see Figure Supplementary Figure 29).

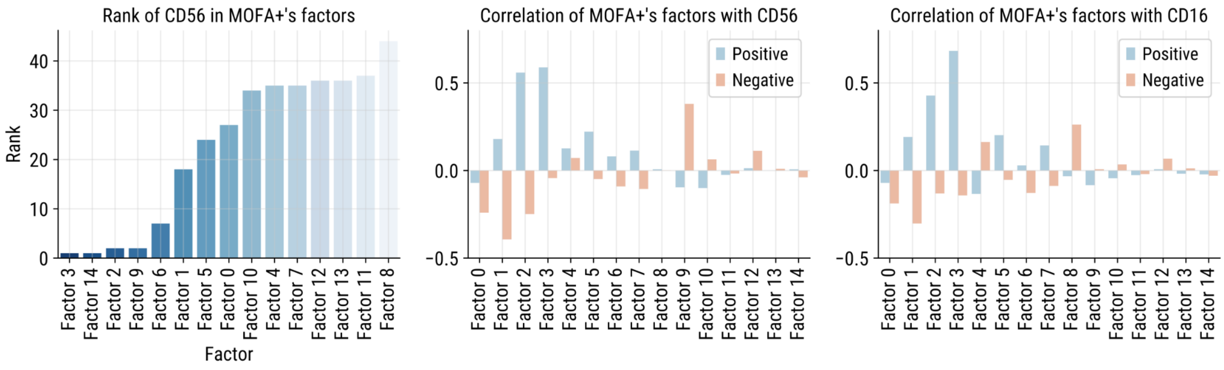

Supplementary Figure 29. Correlation between CD56, CD16, and MOFA+’s factors. Compared rank of CD56 in MOFA+’s factors, and correlation of CD56 and CD16 with MOFA+’s factors.

Instead, MOFA+'s factors where CD56 has a good rank or factors highly correlated with CD56 coincide with general CD56+ populations: NK cells (MOFA+'s factors 2 and 3, positive weights) and MAIT T-cells (MOFA+'s factor 9, negative weights) (see Supplementary Figure 30).

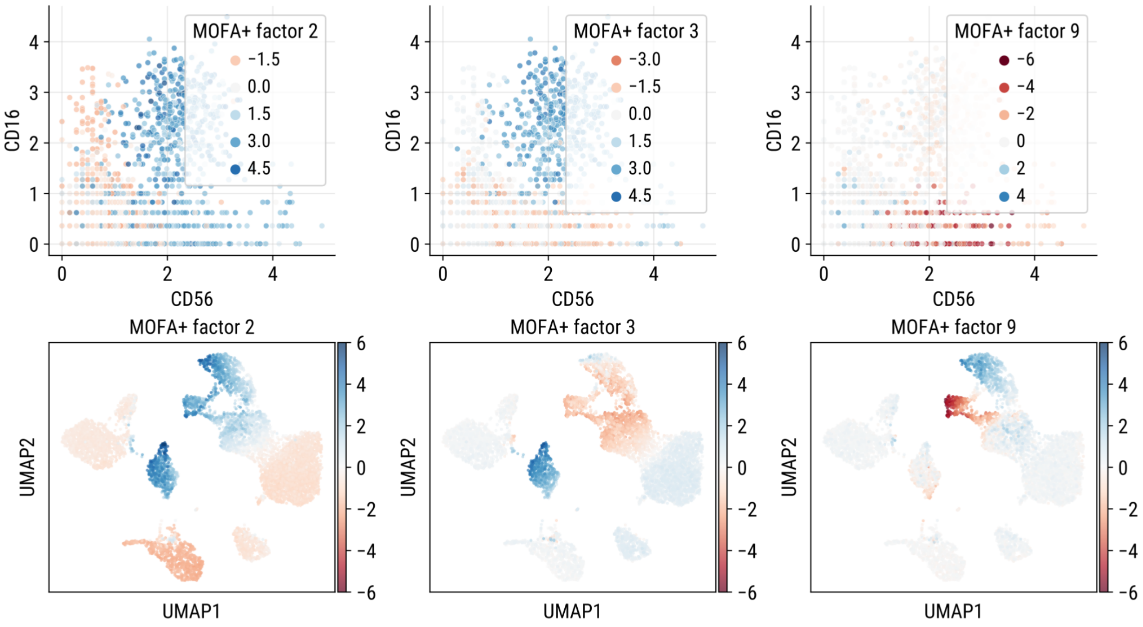

Supplementary Figure 30. Compared ADT profiles and MOFA+ factors. Compared values of CD16, CD56, and weights of MOFA+'s factors 2, 3, and 9.

In conclusion, Mowgli's factor 13 is coherent with the biological signature of CD56 bright NK cells and allows one to distinguish CD56 bright NK cells easily. This is impossible using a unique factor from MOFA+. Indeed, MOFA+'s factors match broad CD56+ populations at best.

2. Plasmacytoid dendritic cells

Plasmacytoid dendritic cells (pDC) have a distinctive CD304-positive and CD14-negative phenotype [6]. Mowgli's factor 3 is largely driven by CD304, and has a low contribution of CD14, and is thus coherent with the biological signature of pDCs (see Supplementary Figure 31).

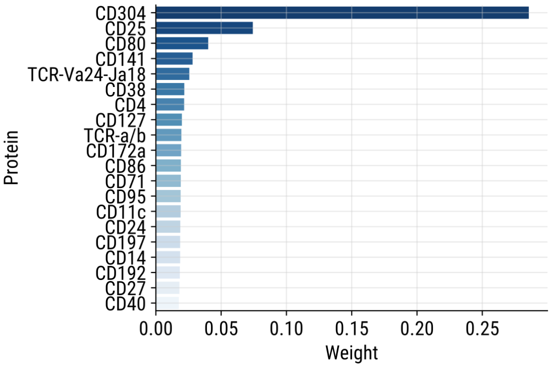

Supplementary Figure 31. Protein weights for Mowgli's factor 3. Top ADT weights for Mowgli's factor 3 in the TEA dataset.

High weights for Mowgli's factor 3 coincide with high CD304 (see Supplementary Figure 32).

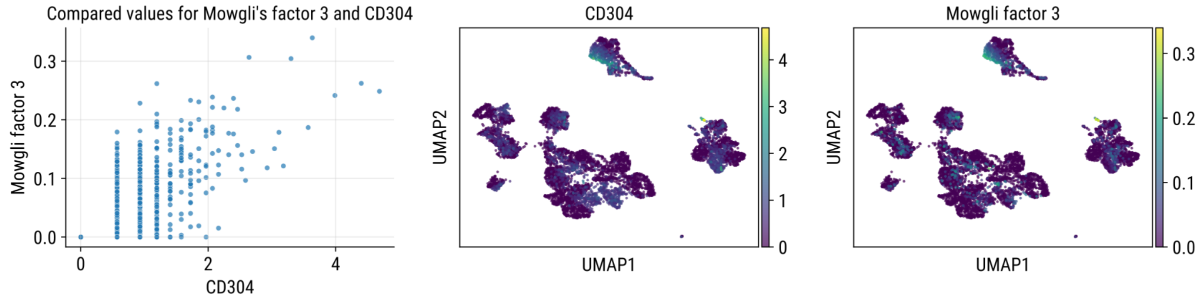

Supplementary Figure 32. Compared ADT profiles and Mowgli factor 3. Compared values of CD304 and weight of Mowgli's factor 3.

In the coarse B-cells cluster, cells with high weights of Mowgli's factor 3 have low CD14 and high CD304, thus corresponding to the pDC phenotype. In the coarse Monocytes cluster, cells with high weights of Mowgli's factor 3 have higher levels of CD14 and lower levels of CD304 than in the B-cells cluster, thus suggesting a CD304+ monocyte subpopulation rather than pDCs (see Supplementary Figure 33). Note also that the highest values of Mowgli's factor 3 are achieved in the coarse B-cells cluster.

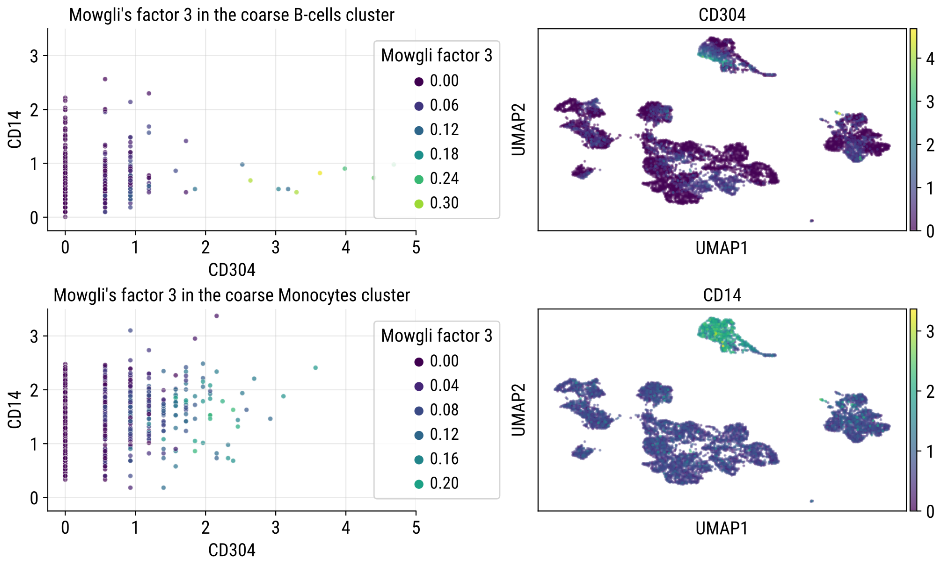

Supplementary Figure 33. Compared ADT profiles and Mowgli factor 3 in subtypes. Compared values of CD304, CD14, and weight of Mowgli's factor 3 in the coarse B-cells and Monocytes subclusters.

Mowgli's factor 3 is the factor most correlated with CD304, and CD304 ranks first among its top proteins (see Supplementary Figure 34).

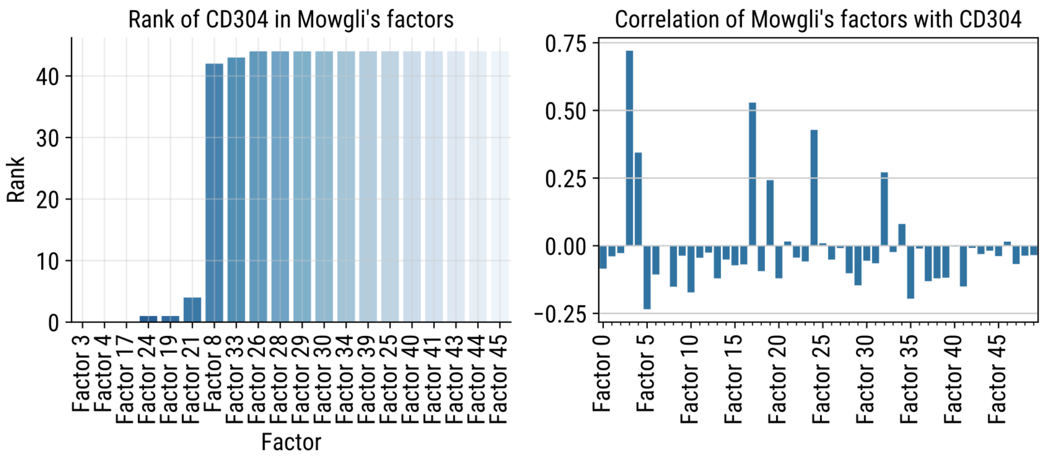

Supplementary Figure 34. Correlation between CD304 and Mowgli's factors. Compared rank of CD304 in Mowgli's factors, and correlation of CD304 with Mowgli's factors.

By contrast, MOFA+ has no factors highly correlated with CD304 or factors where CD304 has a low rank (see Supplementary Figure 35).

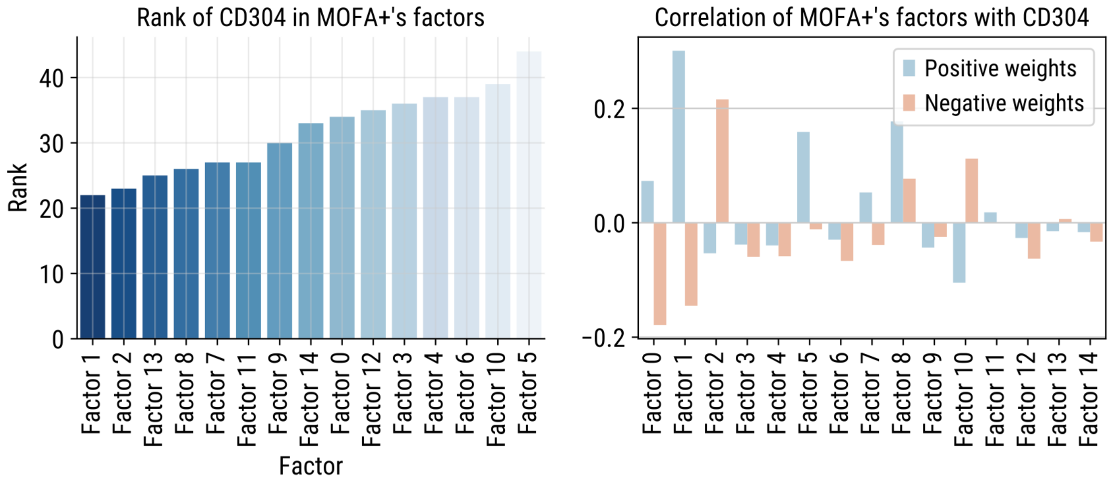

Supplementary Figure 35. Correlation between CD304 and MOFA+'s factors. Compared rank of CD304 in MOFA+'s factors, and correlation of CD304 with MOFA+'s factors.

MOFA+'s factor 1 is the most highly correlated with CD304 and the one where CD304 ranks best, but it coincides with monocytes rather than pDCs (see Supplementary Figure 36).

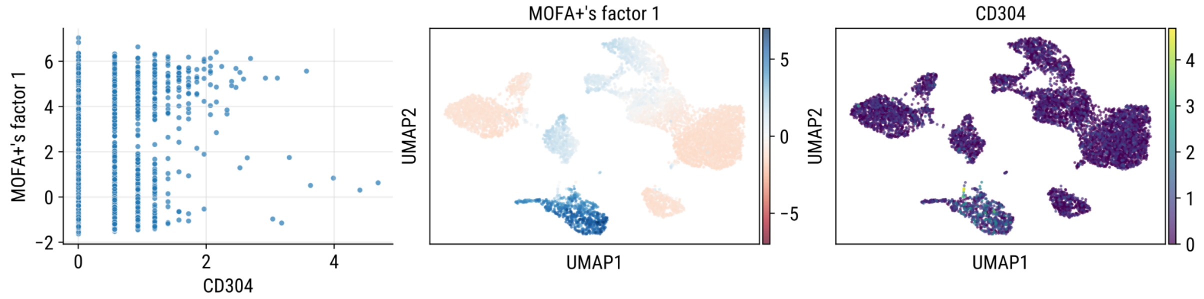

Supplementary Figure 36. Compared ADT profiles and MOFA+ factor 1. Compared values of CD304 and weights of MOFA+'s factor 1.

In conclusion, Mowgli's factor 3 allows one to identify pDCs easily, which is impossible with a unique factor from MOFA+. Indeed, MOFA+ presents no factor closely associated with pDCs, and the best candidate coincides with the broad population of monocytes instead.

3. Naive CD4 T cells

Naive CD4 T cells are CD4-positive and present higher levels of CD45RA than CD45RO. Mowgli's factor 8 is driven by CD4 and has a higher weight for CD45RA than CD45RO (see Supplementary Figure 37).

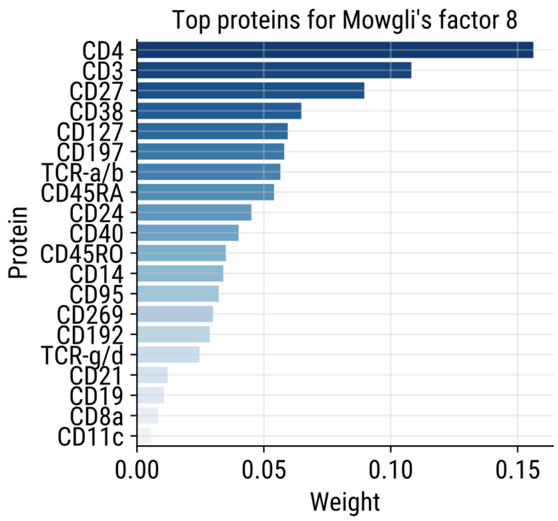

Supplementary Figure 37. Protein weights for Mowgli's factor 8. Top ADT weights for Mowgli's factor 8 in the TEA dataset.

High weights for Mowgli's factor 8 coincide with high CD4, high CD45RA, and low CD45RO (see Supplementary Figure 38).

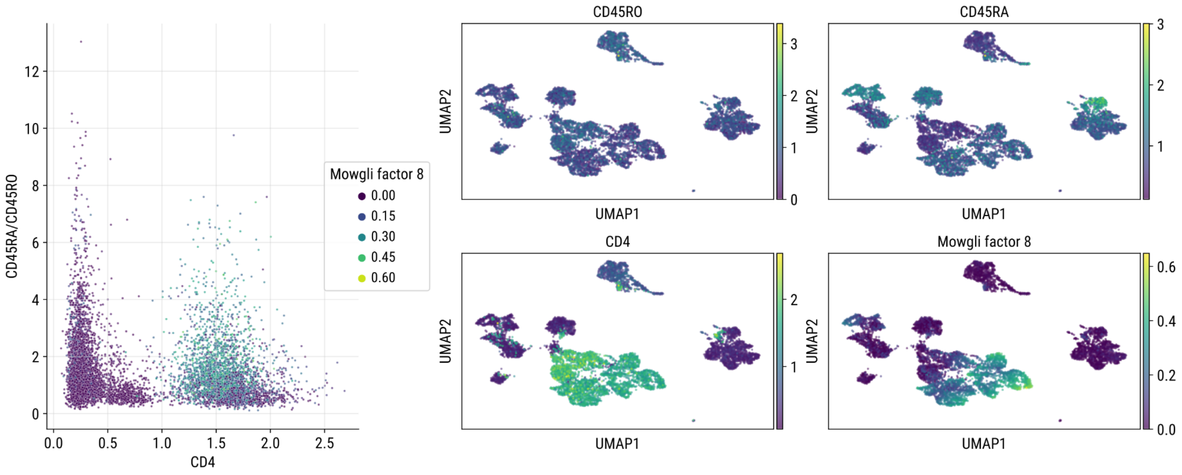

Supplementary Figure 38. Compared ADT profiles and Mowgli factor 8. Compared values of CD4, CD45RA/CD45RO, and weight of Mowgli's factor 8.

CD4 ranks first among the top proteins of Mowgli's factor 8, and factor 8 also has the highest Pearson correlation with CD4 (see Supplementary Figure 39).

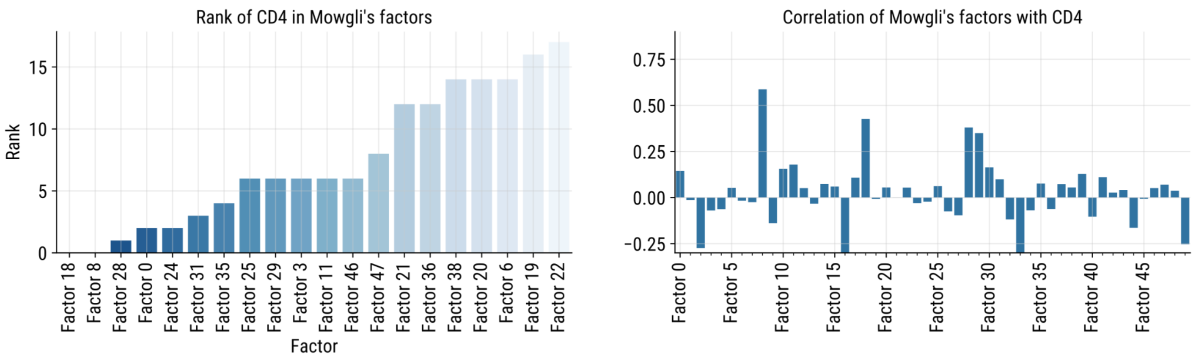

Supplementary Figure 39. Correlation between CD4 and Mowgli's factors. Compared rank of CD4 in Mowgli's factors, and correlation of CD4 with Mowgli's factors.

MOFA+'s factor with the lowest rank for CD4 (factor 7) does not correspond to a CD4 T cell subset. In particular, it does not allow to distinguish CD45RA+CD4+ T cells from CD45RO+CD4+ T cells. MOFA+'s factor most highly correlated with CD4 (factor 0, negative weights) coincides with the general T cell and NK cell populations instead (see Supplementary Figures 40, 41).

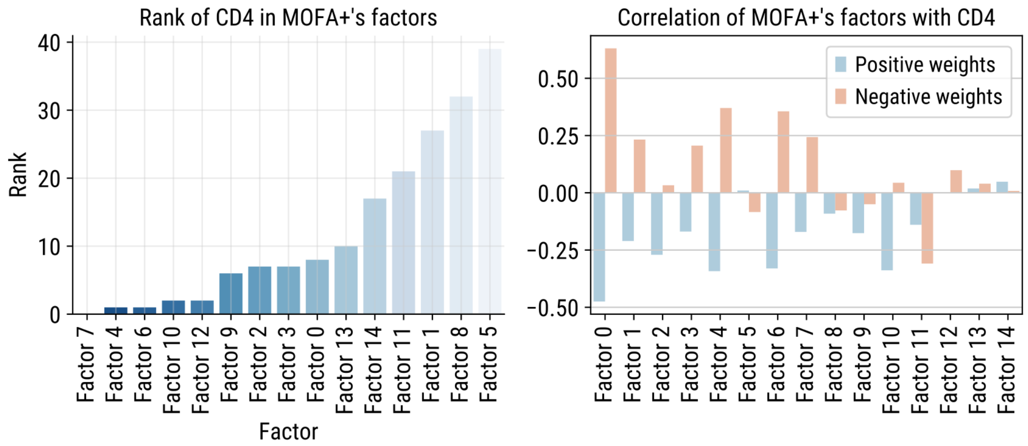

Supplementary Figure 40. Correlation between CD4 and MOFA+'s factors. Compared rank of CD4 in MOFA+'s factors, and correlation of CD4 with MOFA+'s factors.

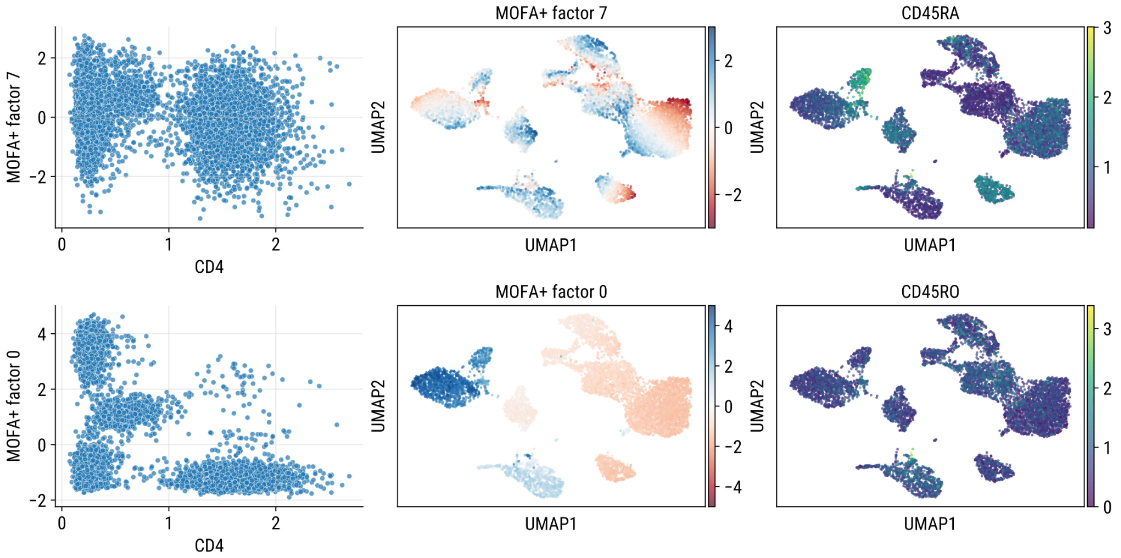

Supplementary Figure 41. Compared ADT profiles and MOFA+ factors 3 and 7. Compared values of CD4, CD45RA, CD45RO, and weights of MOFA+'s factors 0 and 7.

In conclusion, Mowgli's factor 8 allows one to distinguish naive CD4 T cells easily, which is not possible with a unique factor from MOFA+.

4. Conventional dendritic cells

Conventional dendritic cells (cDC) have a distinctive FceRI phenotype. Mowgli's factor 15 is largely driven by FceRI (see Supplementary Figure 42).

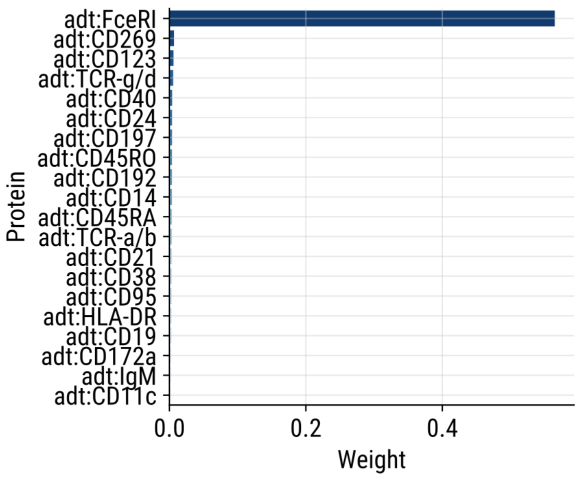

Supplementary Figure 42. Protein weights for Mowgli's factor 15. Top ADT weights for Mowgli's factor 15 in the TEA dataset.

High weights for Mowgli's factor 15 coincide with high FceRI (see Supplementary Figure 43).

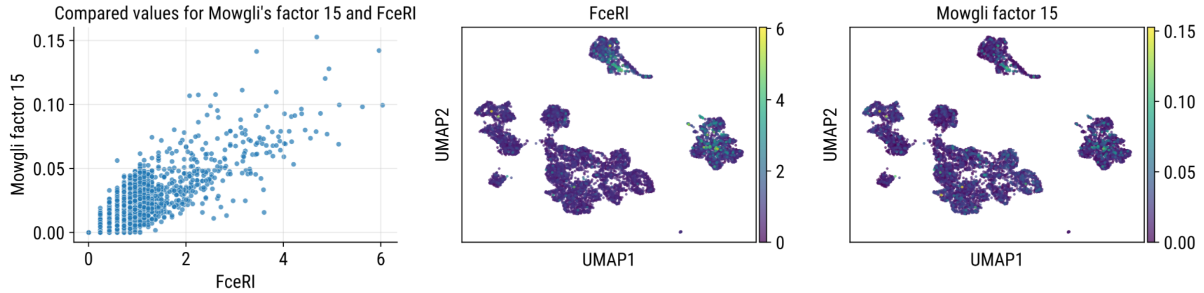

Supplementary Figure 43. Compared ADT profiles and Mowgli factor 15. Compared values of FceRI and weight of Mowgli's factor 15.

Mowgli's factor 15 is the only factor with a high Pearson correlation with FceRI. In addition, FceRI ranks first among factor 15's top proteins, and factor 15 is the only one with a low rank of FceRI (see Supplementary Figure 44).

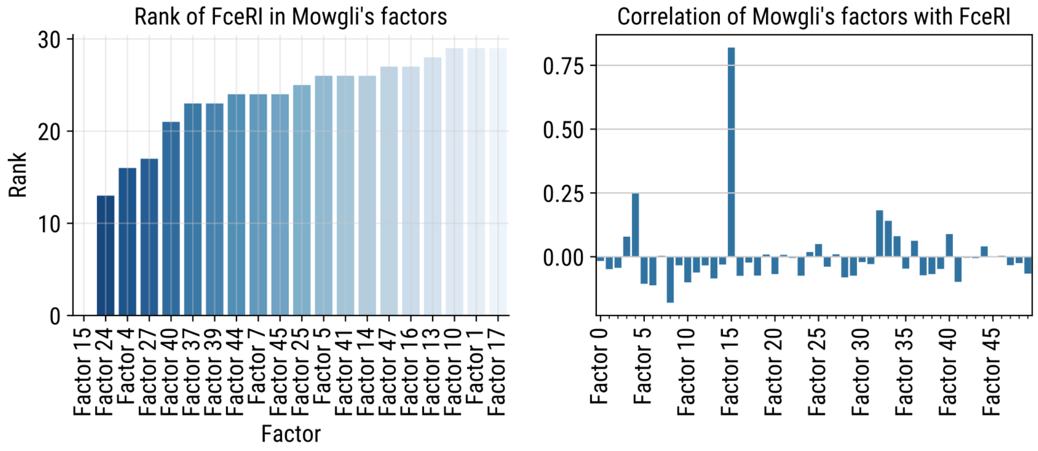

Supplementary Figure 44. Correlation between FceRI and Mowgli's factors. Compared rank of FceRI in Mowgli's factors, and correlation of FceRI with Mowgli's factors.

By contrast, MOFA+ has no factors highly correlated with FceRI or with a low rank of FceRI (see Figure Supplementary Figure 45).

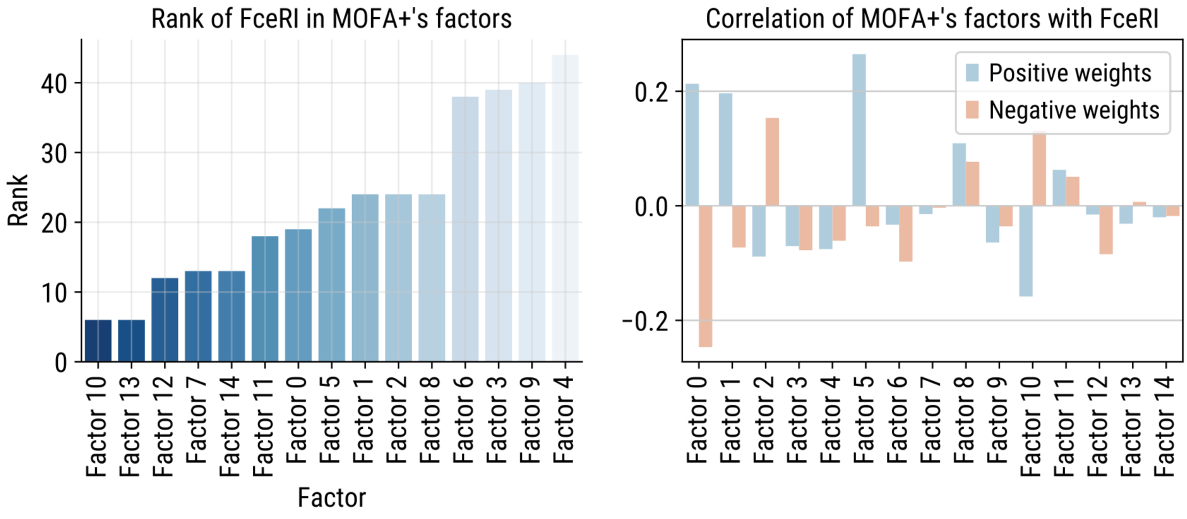

Supplementary Figure 45. Correlation between FceRI and MOFA+'s factors. Compared rank of FceRI in MOFA+'s factors, and correlation of FceRI with MOFA+'s factors.

Even for MOFA+'s factors with the lowest rank for FceRI (factors 10 and 13) or the highest correlation with FceRI (factors 0 and 5), a scatterplot shows no pattern of association between the factor and the cDC phenotype (see Supplementary Figure 46).

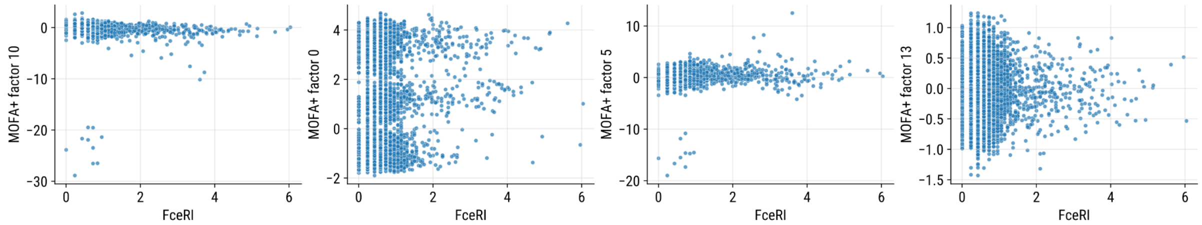

Supplementary Figure 46. Compared ADT profiles and MOFA+ factors 0, 5, 10 and 13. Compared values of FceRI and weights of MOFA+'s factors 0, 5, 10, and 13.

In conclusion, Mowgli's factor 15 allows distinguishing cDCs easily, which is impossible using a unique factor from MOFA+.

5. Nonclassical monocytes

Non-classical monocytes are CD14-low and CD16-high. Mowgli's factor 34 is driven by CD172a and CD11c, proteins present on the surface of monocytes, and has higher weights for CD16 than CD14 (see Supplementary Figure 47).

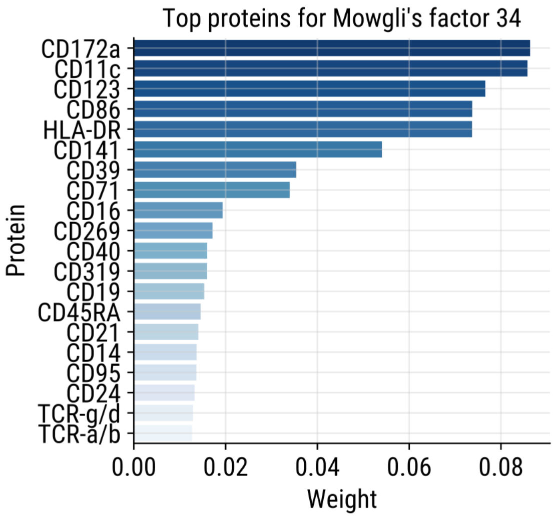

Supplementary Figure 47. Protein weights for Mowgli's factor 34. Top ADT weights for Mowgli's factor 34 in the TEA dataset.

High weights for Mowgli's factor 34 coincide with high CD11c, high CD16, and low CD14 (see Supplementary Figure 48).

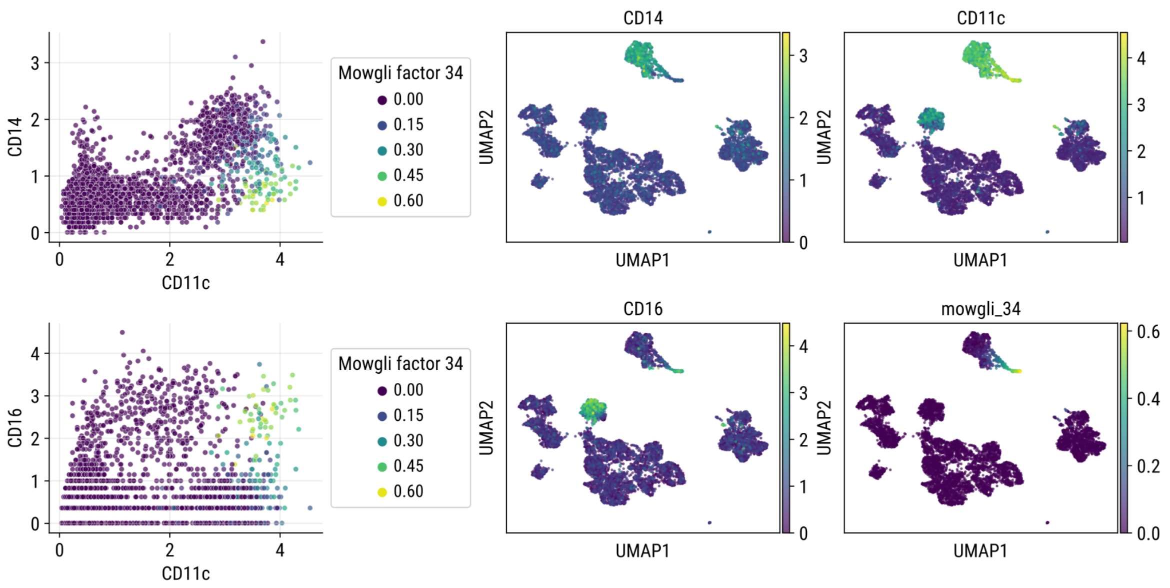

Supplementary Figure 48. Compared ADT profiles and Mowgli factor 34. Compared values of CD14, CD16, CD11c, and weight of Mowgli's factor 34.

MOFA+'s factor 8 has CD16 and CD11c among the top proteins, and CD14's contribution has an opposite sign to CD16 (see Supplementary Figure 49).

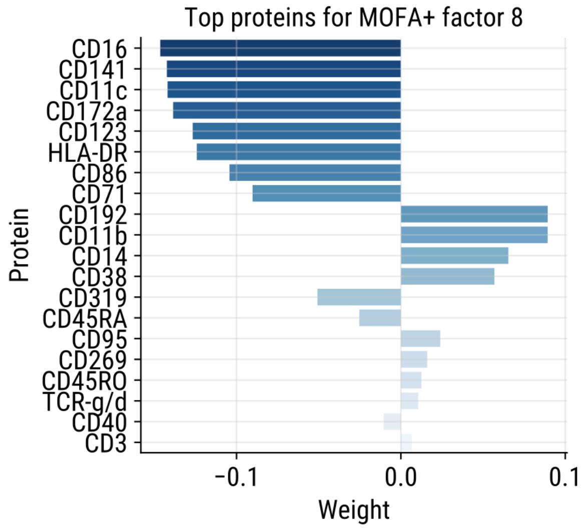

Supplementary Figure 49. Protein weights for MOFA+'s factor 8. Top absolute ADT weights for MOFA+'s factor 8.

Negative weights for MOFA+'s factor 8 coincide with high CD11c, high CD16, and low CD14 (see Supplementary Figure 50).

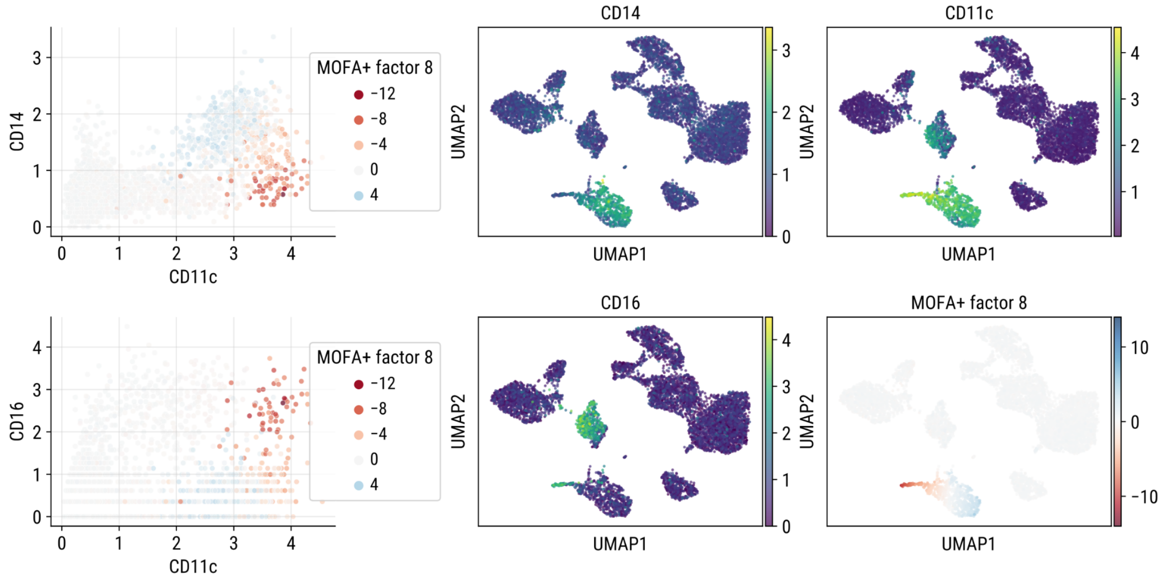

Supplementary Figure 50. Compared ADT profiles and MOFA+ factor 8. Compared values of CD14, CD16, CD11c, and weights of MOFA+'s factor 8.

In conclusion, both Mowgli's factor 13 and MOFA+'s factor 8 allow one to distinguish nonclassical monocytes from classical monocytes.

6. Classical monocytes

Classical monocytes are CD14-positive and CD16-negative. Mowgli's factor 32 is driven by CD172a, CD11b, and CD11c (proteins present on the surface of classical monocytes) and has higher weights for CD14 than CD16 (see Supplementary Figure 51).

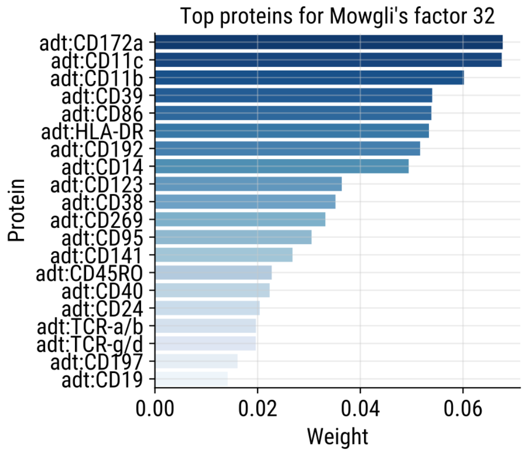

Supplementary Figure 51. Protein weights for Mowgli's factor 32. Top ADT weights for Mowgli's factor 32 in the TEA dataset.

High weights for Mowgli's factor 32 coincide with high CD11c, high CD14, and low CD16 (see Supplementary Figure 52).

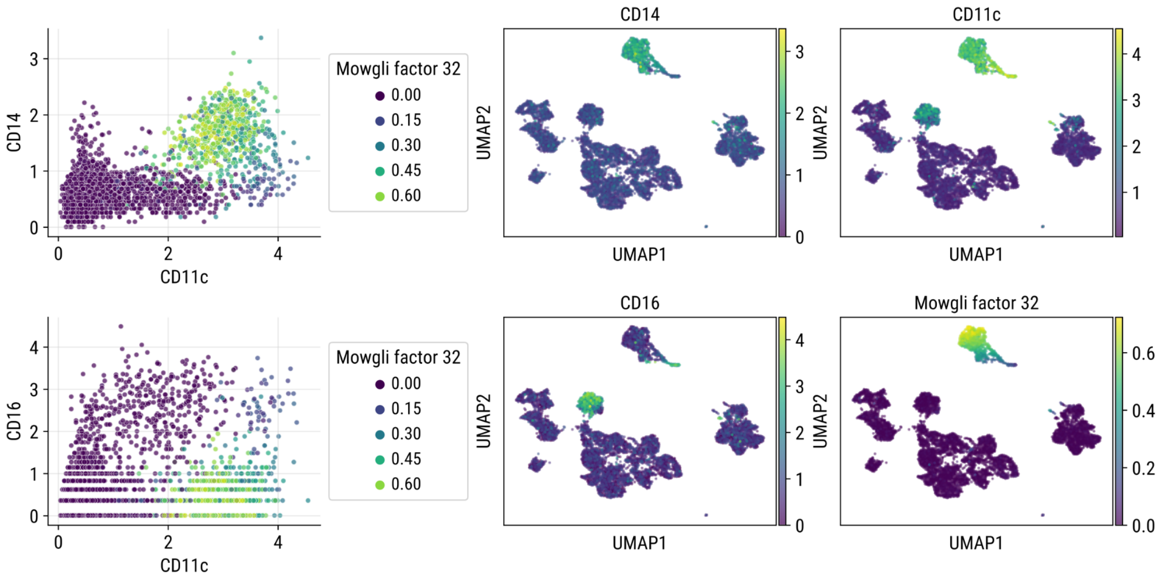

Supplementary Figure 52. Compared ADT profiles and Mowgli factor 32. Compared values of CD14, CD16, CD11c, and weight of Mowgli's factor 32.

CD14 ranks fifth among the top proteins for Mowgli's factor 32, and factor 32 has the highest Pearson correlation with CD14. In addition, factor 32 has one of the lowest absolute Pearson correlations with CD16 (see Supplementary Figure 53).

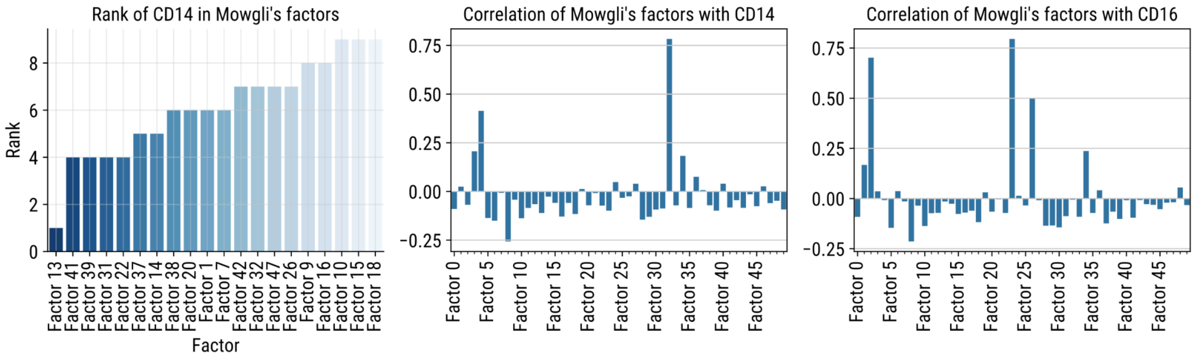

Supplementary Figure 53. Correlation between CD14, CD16, and Mowgli's factors. Compared rank of CD14 in Mowgli's factors, and correlation of CD14 and CD16 with Mowgli's factors.

MOFA+'s factors 1 and 8 have the lowest rank for CD14. They are the most highly correlated with CD14 and have a low correlation with CD16 (see Supplementary Figure 54).

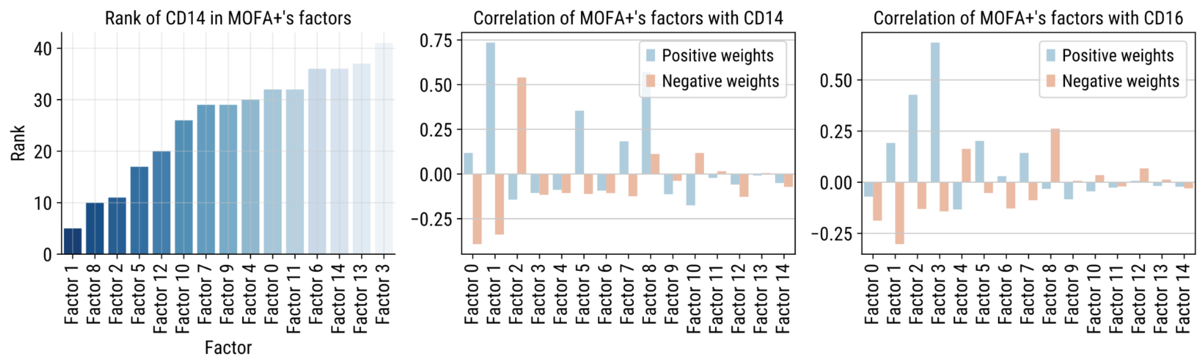

Supplementary Figure 54. Correlation between CD14, CD16, and MOFA+'s factors.
Compared rank of CD14 in MOFA+'s factors, and correlation of CD14 and CD16 with MOFA+'s factors.

Positive weights for MOFA+'s factor 1 coincide with the broad population of monocytes. Positive weights for MOFA+'s factor 8 coincide with classical monocytes (see Supplementary Figure 55). Note that as described previously, negative weights for factor 8 coincide with nonclassical monocytes.

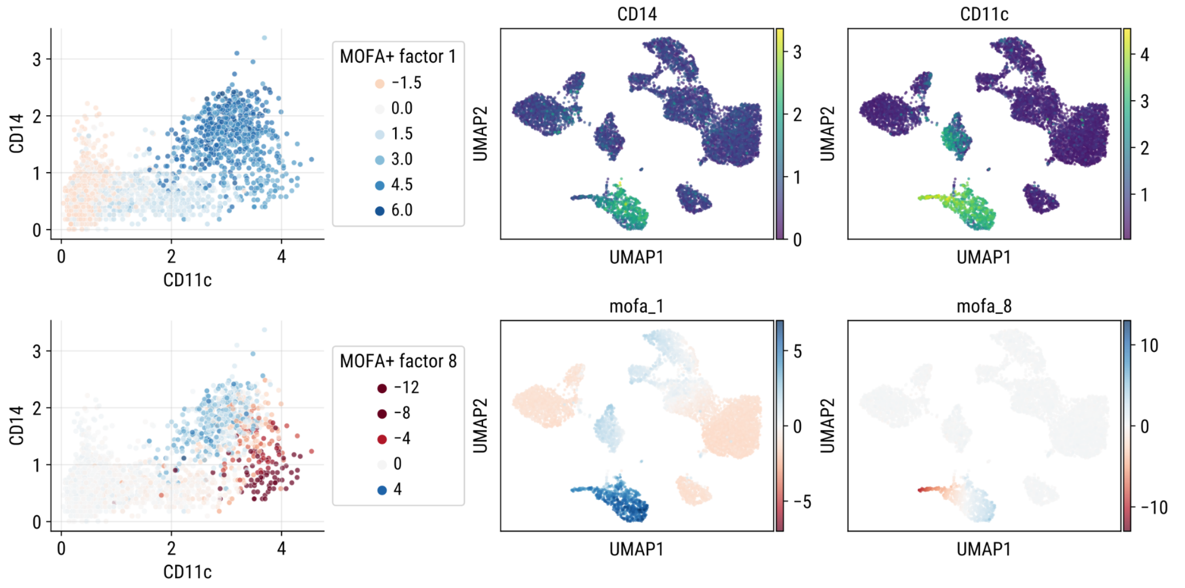

Supplementary Figure 55. Compared ADT profiles and MOFA+ factors 1 and 8.
Compared values of CD14, CD11c, and weights of MOFA+'s factors 1 and 8.

While factor 8 correctly splits nonclassical and classical monocytes, positive weights for MOFA+'s factor 8 do not reflect the biological signature of classical monocytes. Indeed, factor 8 has negative weights for CD11c and CD172a (see Supplementary Figure 56), even though these proteins are present on the surface of both classical and nonclassical monocytes.

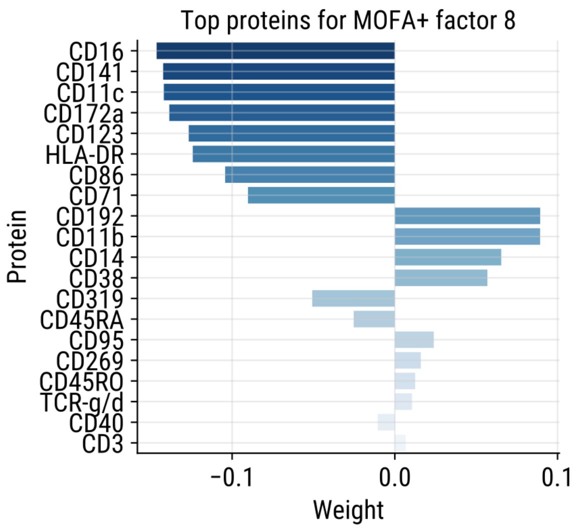

Supplementary Figure 56. Protein weights for MOFA+'s factor 8. Top absolute ADT weights for MOFA+'s factor 8.

In conclusion, MOFA+'s factor 8 allows one to distinguish classical monocytes from nonclassical monocytes, but Mowgli's factor 32 is more clearly associated with the biological signature of classical monocytes than MOFA+'s factor 8.

Supplementary References

1. Liu, L. *et al.* Deconvolution of single-cell multi-omics layers reveals regulatory heterogeneity. Nat. Commun. **10**, 470 (2019).
2. Luecken, M. *et al.* A sandbox for prediction and integration of DNA, RNA, and proteins in single cells. in Proceedings of the Neural Information Processing Systems Track on Datasets and Benchmarks (eds. Vanschoren, J. & Yeung, S.) vol. 1 (2021).
3. Stuart, T. *et al.* Comprehensive Integration of Single-Cell Data. Cell **177**, 1888-1902.e21 (2019).
4. Swanson, E. *et al.* Simultaneous trimodal single-cell measurement of transcripts, epitopes, and chromatin accessibility using TEA-seq. eLife **10**, e63632 (2021).
5. Hao, Y. *et al.* Integrated analysis of multimodal single-cell data. Cell **184**, 3573-3587.e29 (2021).
6. Collin, M. *et al.* Human dendritic cell subsets. Immunology **140**(1), 22-30 (2013).
