# Supplementary material for: Paired single-cell multi-omics data integration with Mowgli
Source: Nat Commun. 2023 Nov 24;14:7711. doi: 10.1038/s41467-023-43019-2 (PMC10673889; doi:10.1038/s41467-023-43019-2)
Supplement: Supplementary file 3 — Reporting Summary [file 41467_2023_43019_MOESM3_ESM.pdf]

nature portfolio

Corresponding author(s): Laura Cantini

Last updated by author(s): Oct 9, 2023

Reporting Summary

Nature Portfolio wishes to improve the reproducibility of the work that we publish. This form provides structure for consistency and transparency in reporting. For further information on Nature Portfolio policies, see our Editorial Policies and the Editorial Policy Checklist.

Statistics

For all statistical analyses, confirm that the following items are present in the figure legend, table legend, main text, or Methods section.

n/a	Confirmed	
☒	☐	The exact sample size (*n*) for each experimental group/condition, given as a discrete number and unit of measurement
☒	☐	A statement on whether measurements were taken from distinct samples or whether the same sample was measured repeatedly
☒	☐	The statistical test(s) used AND whether they are one- or two-sided<br>*Only common tests should be described solely by name; describe more complex techniques in the Methods section.*
☒	☐	A description of all covariates tested
☒	☐	A description of any assumptions or corrections, such as tests of normality and adjustment for multiple comparisons
☒	☐	A full description of the statistical parameters including central tendency (e.g. means) or other basic estimates (e.g. regression coefficient) AND variation (e.g. standard deviation) or associated estimates of uncertainty (e.g. confidence intervals)
☒	☐	For null hypothesis testing, the test statistic (e.g. *F*, *t*, *r*) with confidence intervals, effect sizes, degrees of freedom and *P* value noted<br>*Give P values as exact values whenever suitable.*
☒	☐	For Bayesian analysis, information on the choice of priors and Markov chain Monte Carlo settings
☒	☐	For hierarchical and complex designs, identification of the appropriate level for tests and full reporting of outcomes
☒	☐	Estimates of effect sizes (e.g. Cohen's *d*, Pearson's *r*), indicating how they were calculated

Our web collection on statistics for biologists contains articles on many of the points above.

Software and code

Policy information about availability of computer code

Data collection	No software was used for data collection
Data analysis	Only open-source software was used to perform our experiments.<br>TorchNMF (v0.3.4), MOFA+ (v1.7.3), Seurat (v4), Multigrate (v0.0.2), Cobolt (v1.0.1), and our package Mowgli (v0.1.0) were used to analyse the data.<br>Matplotlib (v3.7.0) and Seaborn (v0.12.2) were used for visalization.<br>Scanpy (v1.9.2) and Muon (v0.1.2) were used for data loading and preprocessing.<br>Signac (v1.9.0) and JASPAR2022 (v0.99.7) were used for motif enrichment analysis.<br>gProfiler (v1.2.2) was used for gene set enrichment analysis, using gene sets from the Enrichr website.<br>The Azimuth wep app was used to validate cell-type assignment in the TEA-seq dataset.<br>Mowgli is implemented using the PyTorch (v1.13.1) framework.<br>Evaluation metrics were computed using Numpy (v1.24.2) and Scikit-learn (v1.2.1).

For manuscripts utilizing custom algorithms or software that are central to the research but not yet described in published literature, software must be made available to editors and reviewers. We strongly encourage code deposition in a community repository (e.g. GitHub). See the Nature Portfolio guidelines for submitting code & software for further information.

Data

Policy information about availability of data

All manuscripts must include a data availability statement. This statement should provide the following information, where applicable:

- Accession codes, unique identifiers, or web links for publicly available datasets
- A description of any restrictions on data availability
- For clinical datasets or third party data, please ensure that the statement adheres to our policy

Regulatory Circuits. At the time of writing, the Regulatory Circuits website http://ww1.regulatorycircuits.org/ is down. We recovered the data from the mirror http://www2.unil.ch/cbg/regulatorycircuits/FANTOM5_individual_networks.tar .

PBMC. We retrieve a 10X Genomics Multiome (RNA + ATAC) dataset with 9,320 PBMCs. Data is available at https://www.10xgenomics.com/resources/datasets/pbmc-from-a-healthy-donor-granulocytes-removed-through-cell-sorting-10-k-1-standard-2-0-0 .

Liu. We retrieve a scCAT-seq (RNA + ATAC) dataset from Liu et al. with 206 cells from three cancer cell lines (HCT116, HeLa-S3, K562). Data is available in the Supplementary Materials of the original publication.

Simulated data. Controlled settings derived from cell lines data were generated from the Liu dataset and can be reproduced using the provided reproducibility code (see Code availability).

OP-Multiome. We retrieve a Multiome (RNA + ATAC) dataset from the Open Problems challenge and select only the first batch, which contains 6,137 BMMCs. The GEO accession number is GSE194122 and the data is available at https://www.ncbi.nlm.nih.gov/geo/query/acc.cgi?acc=GSE194122 .

OP-CITE. We retrieve a CITE-seq (RNA + ADT) dataset from the Open Problems challenge and select only the first batch, which contains 4,249 BMMCs. The GEO accession number is GSE194122 and the data is available at https://www.ncbi.nlm.nih.gov/geo/query/acc.cgi?acc=GSE194122 .

BM-CITE. We retrieve a CITE-seq (RNA + ADT) dataset from Stuart et al. with 29,803 BMMCs. The GEO accession number is GSE128639 and the data is available at https://www.ncbi.nlm.nih.gov/geo/query/acc.cgi?acc=GSE128639 .

PBMC TEA-seq. We retrieve a recent TEA-seq (RNA + ATAC + ADT) dataset from Swanson et al. with 7,084 PBMCs. The GEO accession number is GSE158013 and the data is available at https://www.ncbi.nlm.nih.gov/geo/query/acc.cgi?acc=GSE158013 .

Research involving human participants, their data, or biological material

Policy information about studies with human participants or human data. See also policy information about sex, gender (identity/presentation), and sexual orientation and race, ethnicity and racism.

Reporting on sex and gender	N/A
Reporting on race, ethnicity, or other socially relevant groupings	N/A
Population characteristics	N/A
Recruitment	N/A
Ethics oversight	N/A

Note that full information on the approval of the study protocol must also be provided in the manuscript.

Field-specific reporting

Please select the one below that is the best fit for your research. If you are not sure, read the appropriate sections before making your selection.

☒ Life sciences ☐ Behavioural & social sciences ☐ Ecological, evolutionary & environmental sciences

For a reference copy of the document with all sections, see nature.com/documents/nr-reporting-summary-flat.pdf

Life sciences study design

All studies must disclose on these points even when the disclosure is negative.

Sample size	Our research relies on several public single-cell omics datasets of cell lines and immune cells. The size of these datasets ranges from 206 to 29,803 cells (see Data availability). In each dataset the number of cells is sufficient to represent the cell-type heterogeneity, as demonstrated in the original publications (see Data availability). Sample-size calculation is not applicable in this case, as we did not compare results based on statistical testing, e.g. T-test.

Data exclusions	In the case of the OP-CITE and OP-Multiome datasets, only the first batch was selected to avoid batch effects. The choice is thus unbiased
Replication	Since public datasets have been used, data replicates do not apply to our analysis. Regarding reproduction of code and results, we provide all the code needed to reproduce our experiments and generate our figures in a dedicated Github repository: https://github.com/cantinilab/mowgli_reproducibility<br>The method itself is implemented in a Python package and its code is hosted at https://github.com/cantinilab/Mowgli
Randomization	Each dataset was analysed independently, and the evaluation of our method compared to the state-of-the art did not require any experimental groups.
Blinding	Blinding is not relevant to our study because we evaluated our method on public datasets and our evaluation metrics require no experimental groups.

Reporting for specific materials, systems and methods

We require information from authors about some types of materials, experimental systems and methods used in many studies. Here, indicate whether each material, system or method listed is relevant to your study. If you are not sure if a list item applies to your research, read the appropriate section before selecting a response.

Materials & experimental systems

n/a	Involved in the study
☒	☐ Antibodies
☒	☐ Eukaryotic cell lines
☒	☐ Palaeontology and archaeology
☒	☐ Animals and other organisms
☒	☐ Clinical data
☒	☐ Dual use research of concern
☒	☐ Plants

Methods

n/a	Involved in the study
☒	☐ ChIP-seq
☒	☐ Flow cytometry
☒	☐ MRI-based neuroimaging
